# CYLD regulates cell ferroptosis through Hippo/YAP signaling in prostate cancer progression

**DOI:** 10.1038/s41419-024-06464-5

**Published:** 2024-01-22

**Authors:** Yanan Gu, Shiqi Wu, Junjie Fan, Zeji Meng, Guoqiang Gao, Tianjie Liu, Qi Wang, Huayu Xia, Xinyang Wang, Kaijie Wu

**Affiliations:** 1 Department of Urology, First Affiliated Hospital of Xi’an Jiaotong University, Xi’an, 710061 P. R. China; 2 Assisted Reproduction Center, Northwest Women and Children’s Hospital, Xi’an, 710061 P. R. China; 3 Department of Vascular Surgery, First Affiliated Hospital of Xi’an Jiaotong University, Xi’an, 710061 P. R. China; 4 Department of Urology, Baoji Central Hospital, Baoji, 721008 P. R. China

**Keywords:** Prostate cancer, Cell death

## Abstract

Prostate cancer (PCa) is one of the most common malignancy in men. However, the molecular mechanism of its pathogenesis has not yet been elucidated. In this study, we demonstrated that CYLD, a novel deubiquitinating enzyme, impeded PCa development and progression via tumor suppression. First, we found that CYLD was downregulated in PCa tissues, and its expression was inversely correlated with pathological grade and clinical stage. Moreover, we discovered that CYLD inhibited tumor cell proliferation and enhanced the sensitivity to cell ferroptosis in PCa in vitro and in vivo, respectively. Mechanistically, we demonstrated that CYLD suppressed the ubiquitination of YAP protein, then promoted ACSL4 and TFRC mRNA transcription. Then, we demonstrated that CYLD could enhance the sensitivity of PCa xenografts to ferroptosis in vivo. Furthermore, we discovered for the first time that there was a positive correlation between CYLD expression and ACSL4 or TFRC expression in human PCa specimens. The results of this study suggested that CYLD acted as a tumor suppressor gene in PCa and promoted cell ferroptosis through Hippo/YAP signaling.

## Introduction

Prostate cancer (PCa) is one of the most common cancers worldwide and the fifth leading cause of cancer death in men [1]. PCa often remains dormant in the early stage, but gets diagnosed in the late stage or even metastatic stage. In past several years, many therapeutic strategies for PCa have been extensively explored, such as androgen deprivation therapy (ADT), chemotherapy and target therapy for DNA repair. Nevertheless, the foregoing approaches display limited efficacy, and most patients with advanced PCa develop into castration and drug resistance leading to cancer death [2–4].

CYLD, a novel deubiquitinating enzyme, always acts as a tumor suppressor gene in the development and progression of many cancers. Human CYLD locates on chromosome 16q12.1. The C-terminus of the CYLD protein contains a catalytic ubiquitin-specific protease (USP) domain which catalyzes the cleavage of different polyubiquitin connections [5, 6]. It was initially discovered in patients with familial cylindromatosis, and it had genetic mutations or loss of heterozygosity in this disease [7, 8]. Kovalenko et al. have found that CYLD negatively regulated the nuclear factor kappa-light-chain-enhancer of activated B (NF-κB) signaling pathway and identified the interaction between CYLD and NEMO protein [9]. Various studies have reported that CYLD suppressed cancer progression in different types of tumors, such as melanoma and non-small cell lung cancer [10–14]. In the mouse liver cells, specific ablation of CYLD resulted in the activation of JNK and TAK1, and then could lead to a spontaneous hepatocellular carcinoma. Also, CYLD knocking-out macrophages could increase JNK activity through ubiquitinizing TRAF-2 protein, and studies have confirmed that CYLD knocking-out mice were prone to cancers [11, 12]. However, the expression and roles of CYLD in PCa are largely unknown.

Ferroptosis is a new discovered form of programmed cell death which characterized by iron overload and accumulation of lipid peroxidation. It has been reported that ferroptosis inhibits tumor growth. Unlike normal cells, cancer cells accumulate high levels of iron. Also, there is a significant difference between the sensitivity of different types of cancer cells to ferroptosis [15–21]. Some studies have found that Erastin could induce ferroptosis in human PCa cells harboring the RAS gene. Hence, Erastin or RSL3 along with the standard treatment could inhibit the growth and migration of PCa cells in vitro and hinder advanced PCa progression in vivo [16, 22, 23]. The Hippo signaling pathway is an evolutionarily conserved pathway involved in organ development, homeostasis, tissue regeneration and immune regulation [24], which consists a network of signaling molecules mediated by YAP/TAZ. Phosphorylation of YAP/TAZ promotes its cytoplasmic localization, proteasome degradation and then reduces transcriptional activity [25]. Recently, ferroptosis has also been found to be regulated by the Hippo signaling pathway. Wu et al. have found that E-cadherin suppressed ferroptosis by activating NF2 and Hippo signaling pathway in epithelial cells [26]. Also, another study has found that YAP/TAZ activation made cancer cells sensitive to ferroptosis but resistant to apoptosis [27]. Therefore, targeting ferroptosis may have therapeutic potential for tumor cells with YAP/ TAZ activation.

To the best of our knowledge, our study is the first to demonstrate that CYLD expression is significantly lower in PCa than in normal prostate tissues. Also, based on in vitro and in vivo experiments, we proved that CYLD could suppress cell proliferation and enhance the sensitivity to cell ferroptosis via YAP/ACSL4 pathway. The results might provide more understanding about the molecular mechanism of PCa to improve the treatment through targeting ferroptosis.

## Material and methods

### Cell culture and reagents

The human PCa cell lines DU145, LNCaP, PC-3, 22RV1 were purchased from American Type Culture Collection (ATCC, Manassas, USA). Normal human prostate epithelial cell line RWPE-1 was purchased from the Chinese Academy of Sciences Cell Bank of Type Culture Collection (CBTCCCAS, Shanghai, China). C4-2 cell line was a gift from Dr. Jer-Tsong Hsieh in the University of Texas Southwestern Medical Center (Dallas, TX, USA). RWPE-1 cell line was cultured at 37 ˚C and under 5% CO2 atmosphere in K-SFM medium supplemented with 0.05 mg/mL BPE and 5 ng/mL EGF. PCa cell lines were cultured in RPMI-1640 medium supplemented with 10% fetal bovine serum (FBS) at 37 ˚C with 5% CO2.

### Plasmids, siRNA and cell transfection

CYLD shRNA and scramble control were purchased from GeneCopoeia (Guangzhou, China). The sequences for shCYLD-1: ccCACAATTCAGCAGTTGTTA, shCYLD-2: gcGCTGTAACTCTTTAGCATT, shCYLD-3: gcCCAATACCAATGGAAGTAT, respectively. CYLD overexpressing lentivirus was obtained from GeneCopoeia (Guangzhou, China). YAP was knocked-down by siYAP (RIBOBIO, Guangzhou, China). The sequences for siYAP-1: TGCAGTTTTCAGGCTAATACAGA, siYAP-2: CTCAGGAATTGAGAACAATGACG. For stable CYLD or shRNA-mediated transfection, lentiviral particles were generated by HEK 293 T cells and the filtered supernatant was transferred to target PCa cell lines. Stable clones were maintained using puromycin. Transfections of siRNA were performed using Lipofectamine^3000^ Reagent.

### Reagents and antibodies

All antibodies used in this study were listed below: Anti-CYLD (ab137524, abcam), Anti-ACSL4 (ab205199, abcam), Anti-TFRC (ab214039, abcam), Rabbit monoclonal Anti-YAP (ab205270, abcam), Mouse monoclonal Anti-YAP (66900-1-Ig, Proteintech), Anti-GAPDH (92310SF, CST), Anti-ki67 (28074-1-AP, Proteintech), anti-cyclin D1 (WL01435a), anti-SLC7A11 (T57046, abmart), anti-FSP1 (T55799, abmart), anti-GCH1 (MG880265, abmart) and anti-GPX4 (ab262509, abcam).

### Western blot analysis

PCa cell lines indicated were treated by RIPA buffer supplemented with 1% inhibitors cocktail and 1 mmol/L PMSF (Sigma, St Louis, MO) for 20 min, then centrifuged at 13000 g for 15 min at 4 °C. Then, these proteins were separated by SDS-PAGE and transferred to polyvinylidene fluoride membranes. After blocking in Tris-buffered saline with 0.1% Tween 20 and 5% skim milk for 1 h, the membranes were incubated in the primary antibodies indicated overnight at 4 °C. Then washing 3 times with TBST, membranes were incubated with horseradish peroxidase-conjugated secondary antibodies for 1 h at room temperature. Finally membranes were visualized by an ECL chemiluminescent detection system (Pierce, Rockford, IL).

### RNA extraction and real-time quantitative RT-PCR

RNA was isolated using RNAfast 200 reagents (Fastagen Biotechnology, Shanghai, China). cDNAs were reversely transcribed by the RevertAid kit (Takara, CA), and realtime PCR were carried out in an iCycler thermal cycler (BioRad, Hercules, CA) using SYBR Green Supermix (TAKARA, CA). Primers: CYLD, F: TCAGGCTTATGGAGCCAAGAA, R: ACTTCCCTTCGGTACTTTAAGGA; ACSL4, F: CATCCCTGGAGCAGATACTCT, R: TCACTTAGGATTTCCCTGGTCC; TFRC, F: ACCATTGTCATATACCCGGTTCA, R: CAATAGCCCAAGTAGCCAATCAT; YAP, F: TAGCCCTGCGTAGCCAGTTA, R: TCATGCTTAGTCCACTGTCTGT; GPX4, F: GAGGCAAGACCGAAGTAAACTAC, R; CCGAACTGGTTACACGGGAA

FTH1, F: CCCCCATTTGTGTGACTTCAT, R: GCCCGAGGCTTAGCTTTCATT;

VDAC2, F: GGCGTGGAATTTTCAACGTCC, R: AGACCATACTCACACCACTTGTA; AIFM2, F:AGACAGGGTTCGCCAAAAAGA, R: CAGGTCTATCCCCACTACTAGC

18 S, F: GGAATTGACGGAAGGGCACCACC, R:GTGCAGCCCCGGACATC TAAGG. All the experiments were repeated for 5 times.

### Cell viability assay

PCa cell lines were seeded into 96-well plates in a density of ~4000/well. At least 10 h after cell inoculation, the medium was carefully aspirated, then RSL3 or Erastin of different concentrations were added to each well, mixed well and incubated for 14 h at 37 ˚C with 5% CO2. Then the media was removed, and 180 µl fresh media mixed with 20 µl MTT (Sigma-Aldrich; USA) was added to each well for 3 h at 37 ˚C. At last, the medium was removed clearly and 150 µl DMSO was placed into each well. The OD value at 450 nm was measured by microplate spectrophotometer .

### Cell cycle assay

DU145 and PC-3 sublines were seeded into 6-well plates in a density of ~5,00000/well. At least 12 h after cell inoculation, the medium was carefully aspirated. After washing cells twice with PBS solution, we collected the cells into tubes. All cells were treated by the cell cycle kit and then analyzed by flow cytometry.

### Lipid peroxidation and cell death assay

The established PCa cell lines were seeded in 24-well plates. At least 10 h after cell inoculation, the medium was carefully aspirated, then RSL3 or Erastin of different concentrations were added to each well. Cells were treated for the indicated times and 5 µM C11-BODIPY 581/591 was added to measure lipid peroxidation. To measure cell death, the cells (including floating dead cells) were harvested and stained with 5 µg/mL PI. Intracellular lipid peroxidation and cell death levels were measured by flow cytometry.

### GSH assay

The established PCa cell lines were treated by the GSH kit according to the manufacturer’s instructions, and the absorbance was measured at 412 nm. According to calibration curve generated by standard, the GSH of samples was quantified.

### Iron and labile iron pool (LIP) measurement

Intracellular iron was determined by FerroOrange. The established PCa cell lines were incubated with 1 μM FerroOrange for 30 min, and images were captured using a fluorescence microscope. Labile iron pool (LIP) measurement based on calcein dequenching has been used. The established PCa cell lines were seeded into 96-well plates in a density of ~5000/well. At least 8 h after cell inoculation, the medium was carefully aspirated, then 250 nM calcein acetoxymethylester in medium with BSA were add into each cell for 30 min. After washing cells twice by PBS, the absorbance was measured at the excitation wavelength of 468 nm, emission wavelength of 517 nm.

### Co-immunoprecipitation assay

The anti-YAP antibody was added into 200 µl of cell lysate and incubated overnight at 4 ˚C to form immune complexes. Then we transfered the immune complexes solution to a tube containing washed magnetic bead. After incubated 1 h at 37 ˚C, the magnetic beads were precipitated into balls by a magnetic separator in ice. Then the sediments were washed five times using 500 µl 1× cell lysis buffer in ice. After heating 5 min at 100 ˚C, the samples were put on a magnetic separation in order to separate magnetic beads and proteins. These proteins were analyzed by Western blot analysis.

### Immunofluorescence assay

PCa cell lines indicated were seeded into 24-well plates, covered with 4% formaldehyde 30 min at 37 ˚C and then rinsed three times with PBS for 5 minutes each time. After blocking in 5% bovine serum albumin for 1 h at 37 ˚C, the samples were incubated with the primary antibodies overnight at 4 ˚C. Then washing 3 times with PBST, the samples were incubated with fluorescein-conjugated secondary antibody for 1 h at room temperature and stained by DAPI (1:1000) for 20 min in the dark. Laser scanning confocal microscopy (Nikon A1R/A1) was used to observe the samples.

### Subcutaneous xenograft tumour model

Male nu/nu mice (6-8 weeks of age) were used according to protocols approved by the Ethical Committee of Xi’an Jiaotong University. PCa cell lines indicated were resuspended in ice with RPMI-1640 and the same amount of cells were injected into mice subcutaneously. Tumor growth was measured with callipers every three days. For drug treatment experiments, 8 days after tumor cell injection, nude mice were treated with physiological saline, 10 mg/kg Liproxstatin-1 (Sigma, SML1414) or 50 mg/kg IKE (Selleck, PUN30119) through intraperitoneal injection every three days until the endpoint as indicated.

### Clinical specimens and immunohistochemistry (IHC) staining

The PCa tissues chip was purchased from Shanghai Xinchao Company (HProA150CS01, XT19-024), which included 50 adjacent non-tumor tissues and 100 PCa tissues. Also, the prostate biopsies of PCa (*n* = 20) were obtained from the Department of Urology, The First Affiliated Hospital of Xi’an Jiaotong University. IHC staining for clinical specimens and subcutaneous transplantation tumors in mice was carried out by the DAKO EnVision system as described in our previous studies [28]. Sections were deparaffinized, rehydrated, and subjected to antigen retrieval in citrate buffer for 5 min. After dual Block was used for 10 min to block endogenous peroxidase and alkaline phosphatase activities, the slides were incubated in the primary antibodies against CYLD (ab137524, abcam, 1:200), ACSL4 (ab205199, abcam, 1:500), Anti-TFRC (ab214039, abcam, 1:500) or ki67 (28074-1-AP, Proteintech, 1:400) overnight at 4 °C. This step was followed by washing and incubation with secondary antibody for 1 h. Then the slides were stained with a diaminobenzidine (DAB) kit and haematoxylin. The expression of CYLD, ACSL4, TFRC and ki67 was scored based on percentage and intensity according to Allred’s.

### Bioinformatic data and statistical analysis

All samples used for clinical statistical analysis were excavated from The Cancer Genome Atlas (TCGA; https://www.genome.gov/Funded-Programs-Projects/Cancer-Genome-Atlas) and Gene Expression Omnibus (GEO; https://www.ncbi.nlm.nih.gov/geo/) databases (GSE130451, GSE6919) by the standard processing pipeline. The prostate public Statistical analyses were performed using GraphPad Prism version 7.0 (GraphPad Software, CA). All the statistical analyses were performed by SPSS 22.0 software. two-tailed Student’s (t-test) or one-way analysis were used for comparisons as indicated. The Z-score was calculated to normalize RNA expression. *p* value < 0.05 was considered statistically significant.

## Results

### Downregulation of CYLD expression in prostate cancer

**Fig. 1:**
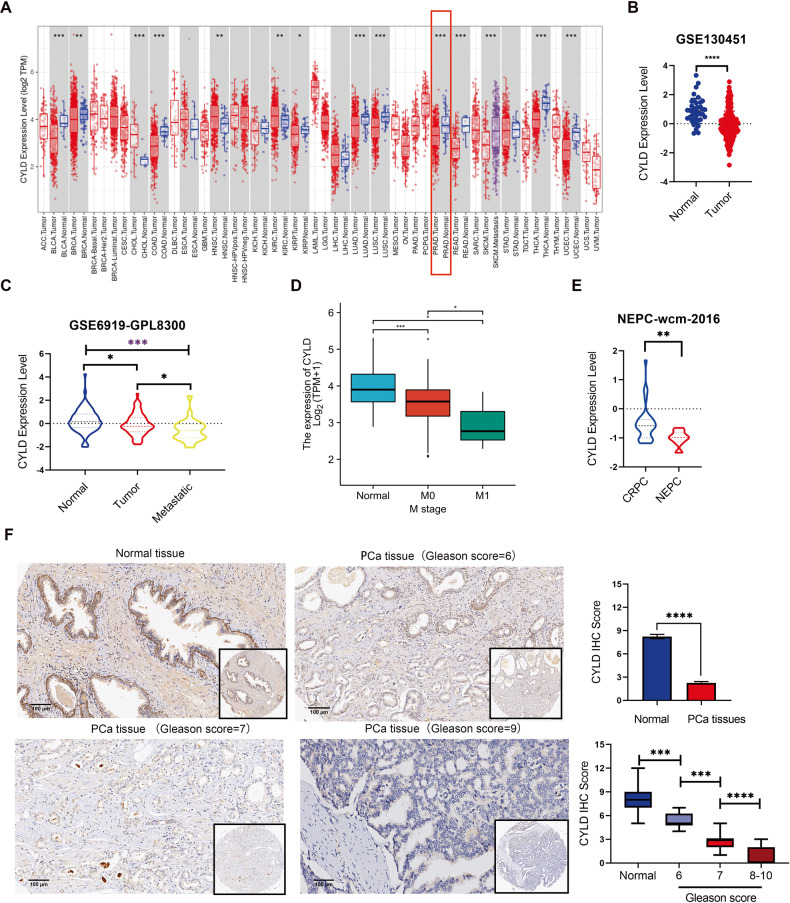
Downregulation of CYLD expression in PCa. **A** CYLD mRNA expression in normal and cancer tissues from TCGA database. **B**. CYLD mRNA expression in normal prostate tissues and PCa tissues from GEO databases (GSE130451). **C**, **D** CYLD mRNA expression in normal prostate tissues, PCa tissues and metastatic PCa tissues from GEO databases (GSE6919-GPL8300) and TCGA database. **E** CYLD mRNA expression in CRPC and NEPC tissues from NEPC-wcm-2016 database. **F** Representative picture of CYLD protein expression in PCa tissue chip detected by IHC and quantification of CYLD protein was shown. Scale bar = 100 μm. (*p < 0.05; **p < 0.01; ***p < 0.001; ****p < 0.0001).

To explore the CYLD mRNA expression level in PCa, we mined the TCGA and GEO databases and found that the expression of CYLD mRNA was lower in PCa tissues compared to prostate tissues in the TCGA and GEO series GSE130451 (Fig. 1A, B). Also, the GEO database series (GSE62293, GSE8511) disclosed that the expression level of CYLD mRNA in PCa was significantly lower than in their paired adjacent normal tissues (supplemental Fig. 1A). In addition, the expression level of CYLD mRNA in metastatic PCa tissues was lower than that in primary PCa tissues based on the analysis of the GSE6919 and TCGA (Fig. 1C, D). Moreover, CYLD mRNA expression was lower in neuroendocrine prostate cancer (NEPC) than in castration-resistant prostate cancer (CRPC) (Fig. 1E). To investigate the protein expression of CYLD in PCa, we performed IHC analysis on the PCa tissue chip. The results showed that the expression of CYLD protein in PCa tissues were reduced compared to normal prostate tissues. Moreover, the expression of CYLD protein in PCa tissues with Gleason score 8-10 was downregulated compared with that in PCa tissues with Gleason score 6-7 (Fig. 1F). Then we explored CYLD deletion in PCa tissues by analyzing data mined from cBioPortal (https://www.cbioportal.org/). We detected deep and shallow deletion of the CYLD gene in 1/136 and 63/136 PCa tissues, respectively (supplemental Fig. 1B). In addition, we sorted out the gene sequencing reports of 5499 PCa patients from eight different domestic hospitals of P.R.China, including the Department of Urology in the First Affiliated Hospital of Xi’an Jiaotong University. Of these cases, we found 421 patients had CYLD gene mutations, and the population mutation rate was about 7.656%. The principal nucleic acid mutations included splicing (82.19%) and deletion (12.83%). The main amino acid mutation was missense (80.05%) (supplemental Fig. 1C). Therefore, our results indicated that CYLD expression decreased due to gene deletion during PCa progression.

### CYLD suppressed PCa cell proliferation in vitro and in vivo

**Fig. 2:**
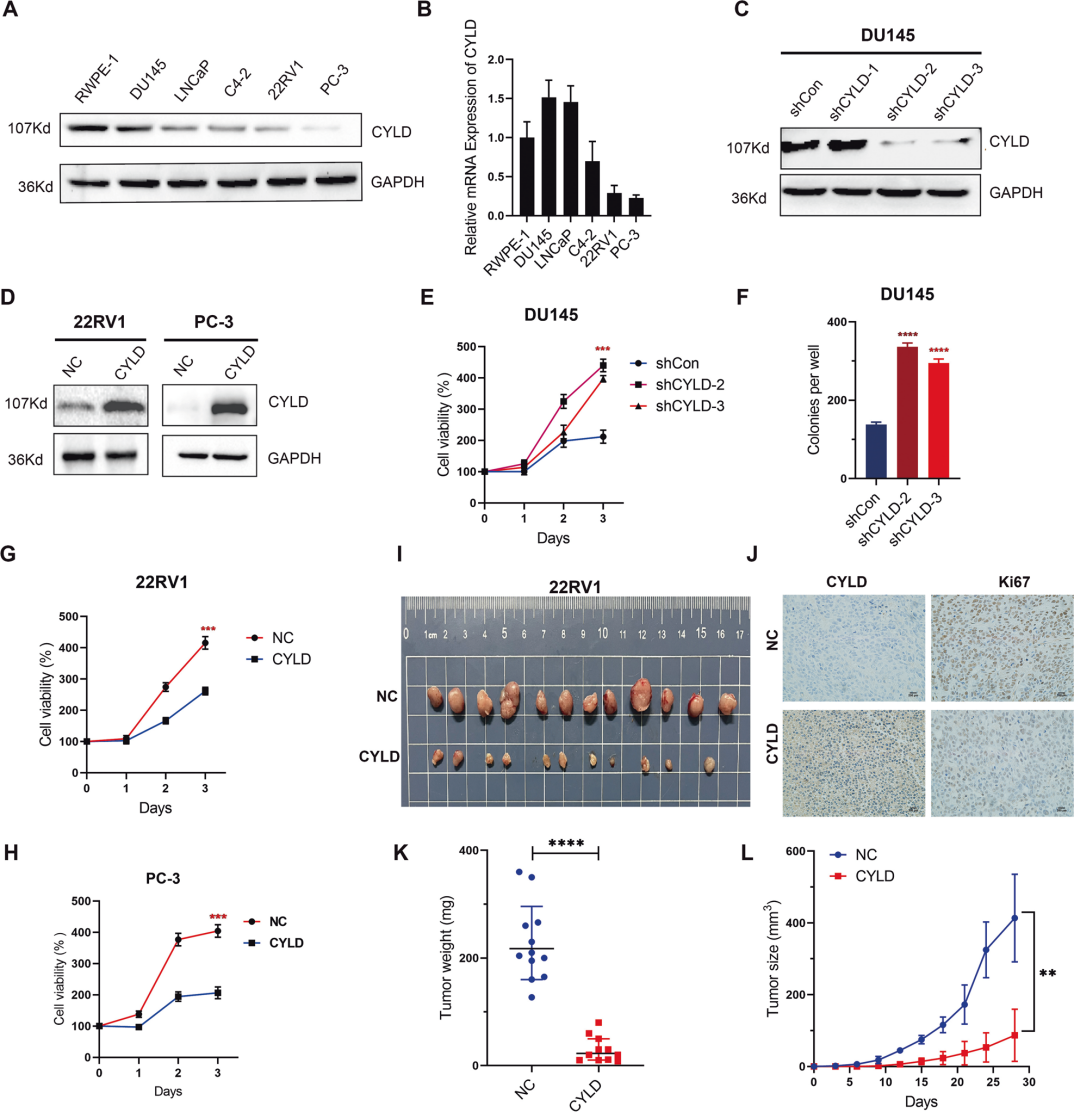
CYLD suppressed PCa cell proliferation in vitro and in vivo. **A** Western blot analysis of CYLD expression levels in human PCa cell lines and the normal prostate cell line RWPE-1. GAPDH was used as the loading control. **B** Quantitative real-time PCR (qRT-PCR) analysis of the CYLD mRNA expression levels in PCa cell lines and the normal prostate cell line. 18 S was applied as the endogenous control. Data are presented as the mean ± SEM, *n* = 3. **C** Western blot analysis of CYLD expression in DU145 cell lines transfected with CYLD shRNAs and shControl. **D** Western blot analysis of CYLD expression in 22RV1 and PC-3 cell lines transfected with CYLD lentivirus and negative control. **E** Cell viability was detected by MTT assay in DU145 cell lines transfected with CYLD shRNAs and shControl. **F** Colony formation of DU145 cell lines transfected with CYLD shRNAs and shControl, and quantitative analysis was shown (*N* = 3). **G** Cell viability was detected by MTT assay in 22RV1 transfected with CYLD lentivirus and negative control. **H** Cell viability was detected by MTT assay in PC-3 transfected with CYLD lentivirus and negative control. **I**, **J**, **K** **L** Images of the tumor growth of subcutaneous xenografts established by CYLD overexpressing 22RV1 sublines. The wet weights of xenografts were measured after harvesting and tumor volumes were measured every three days. IHC analysis of the expression levels of CYLD and Ki67 in xenografts was shown. (**p < 0.01; ***p < 0.001).

To verify the expression of CYLD in PCa cells, we extracted proteins and mRNA from human normal prostate epithelial cell line RWPE-1 and PCa cell lines (DU145, LNCaP, C4-2, 22RV1 and PC-3) for Western blot and RT-PCR experiments, respectively (Fig. 2A, B). The expression of CYLD protein and mRNA was higher in DU145 cells and comparatively lower in 22RV1 and PC-3 cells. Thus, we generated CYLD knocking-down DU145 subline and CYLD overexpressing 22RV1 and PC-3 sublines (Fig. 2C, D). After knocking-down CYLD in PCa cells, the cell proliferation significantly increased by MTT and colony formation assay (Fig. 2E, F). To be the contrary, CYLD over-expression in 22RV1 or PC-3 cells significantly decreased the cell proliferation in vitro and tumorigenesis in vivo (Fig. 2G, H and supplemental Fig. 2A, B). Similarly, after overexpressing CYLD in DU145, the cell proliferation significantly decreased by MTT assay (supplemental Fig. 2C, D). The tumors volume and weight of 22RV1/CYLD groups was significantly lower than 22RV1/NC group, also the IHC analysis of CYLD and Ki67 in the xenograft tissues confirmed this inhibitory effect of CYLD on tumor growth in vivo (Fig. 2I, J, K, L).

### CYLD enhanced the sensitivity of PCa cells to ferroptosis in vitro

**Fig. 3:**
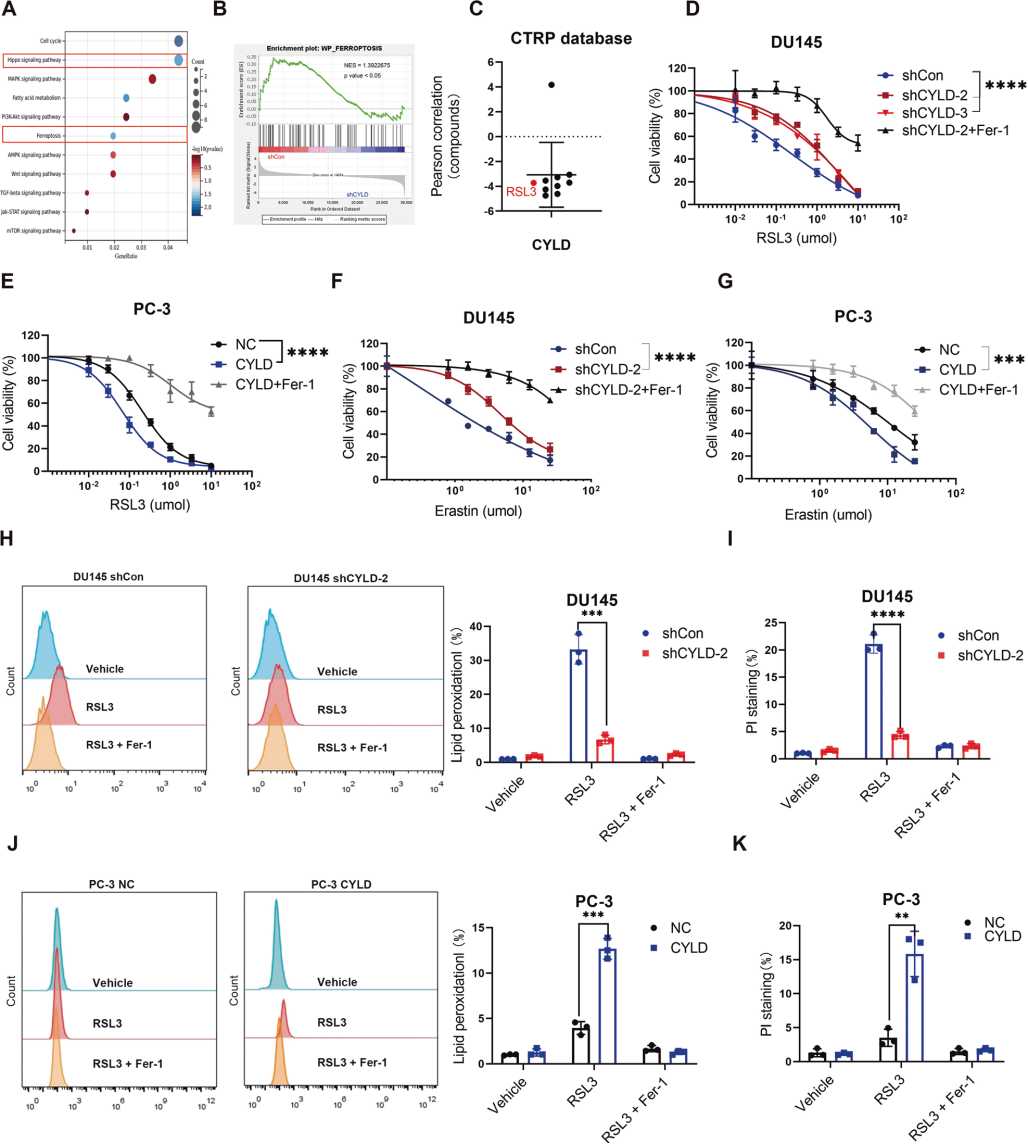
CYLD enhanced the sensitivity of PCa cells to ferroptosis in vitro. **A** Transcriptome high-throughput sequencing (HTS) on samples of DU145 shCon and DU145 shCYLD sublines. **B** GSEA results showed that “WP_ferroptosis” was downregulated in CYLD knocking-down DU145 subline. **C** The negative correlation between the area under the curve (AUC) value for RSL3 and CYLD expression from the CTRP database. **D** Cell viability of DU145/shCYLD and DU145/shCon sublines after RSL3 or ferrostatin-1 (Fer-1; 5 μM) treatment as indicated. **E** Cell viability of PC-3/CYLD and PC-3/NC sublines after RSL3 or ferrostatin-1 (Fer-1; 5 μM) treatment as indicated. **F** Cell viability of DU145/shCYLD and DU145/shCon sublines after Erastin or Ferrostatin-1 (Fer-1; 5 μM) treatment as indicated. **G** Cell viability of PC-3/CYLD and PC-3/NC sublines after Erastin or Ferrostatin-1 (Fer-1; 5 μM) treatment as indicated. **H**. Lipid peroxidation measurement in DU145/shCYLD and DU145/shCon sublines treated with RSL3 for 6 h, and quantitative analysis was shown (*N* = 3). **I** Results of PI staining of DU145/shCYLD and DU145/shCon sublines treated with RSL3 for 12 h. **J** Lipid peroxidation measurement in PC-3/CYLD and PC-3/NC sublines treated with RSL3 for 12 h. **K** Results of PI staining of PC-3/CYLD and PC-3/NC sublines treated with RSL3 for 20 h. (**p < 0.01; ***p < 0.001; ****p < 0.0001).

We conducted transcriptome high-throughput sequencing (HTS) on samples of DU145/shCon and DU145/shCYLD sublines to elucidate the mechanisms by which CYLD inhibited PCa growth. Then we mapped the HTS results to the background set and obtained the gene set enrichment output using R (R Core Team, Vienna, Austria). It was worth noting that Hippo pathway and ferroptosis pathway were significantly enriched (Fig. 3A). Similar results were obtained from the high-throughput sequencing analyses of PC-3/NC and PC-3/CYLD sublines (supplemental Fig. 3A). Given that literature reporting that ferroptosis could inhibit cancer progression and the Hippo pathway was closely associated with ferroptosis, we speculated whether CYLD acted as an inhibitory effect on PCa through ferroptosis. Thus we enriched the ferroptosis signaling pathway collection and found that “WP_Ferroptosis_SIGNALING” was mainly enriched in the shCon group (Fig. 3B). The analysis of Cancer Therapeutics Response Portal (CTRP) databases indicated there was a negative correlation between the area under the curve (AUC) value for RSL3 and CYLD expression and it ranked in the top five. Hence, the resistance of GPX4i was negatively correlated with the expression level of CYLD (Fig. 3C). To validate the role of CYLD in the ferroptosis of PCa cells, we subjected the established PCa cell lines to the class II ferroptosis inducer (RSL3) or the class I ferroptosis inducer (Erastin), then MTT assays were applied to assess the sensitivity of PCa cell lines to these ferroptosis inducers. The results showed that CYLD knockdown in DU145 cells decreased the sensitivity of cancer cells to ferroptosis, while CYLD overexpression in PC-3 cells, 22RV1 and DU145 cells increased the sensitivity of cancer cells to ferroptosis (Fig. 3D–G, and supplemental Fig. 3B, C). Also, cell death and intracellular lipid peroxidation levels were measured by flow cytometry after these established cell lines were treated with a variety of ferroptosis inducers for different time. The results showed that CYLD knockdown in DU145 cells decreased cell death and intracellular lipid peroxidation levels (Fig. 3H, I), while CYLD overexpression in PC-3 and DU145 cells increased cell death and intracellular lipid peroxidation levels (Fig. 3J, K and supplemental Fig. 3D, E). Also, we observed that one of the highest enriched pathways obtained in the RNAseq analysis was cell cycle in these PCa sublines (Supplemental Fig. 3F), which might be associated with ferroptosis susceptibility. Therefore, we further performed flow cytometry to assess the cell cycle distribution of DU145 and PC-3 sublines. As expected, CYLD over-expression in PCa cells suppressed the G1-S phase transition (Supplemental Fig. 3G). Consistently, cyclin D1 was upregulated in the CYLD knocking-down cells, while downregulated in the CYLD overexpressing cells (Supplemental Fig. 3H).

The preceding results indicated that overexpression of CYLD could enhance the sensitivity of PCa cell lines to ferroptosis.

### CYLD interacted with and stabilized YAP protein by deubiquitination

**Fig. 4:**
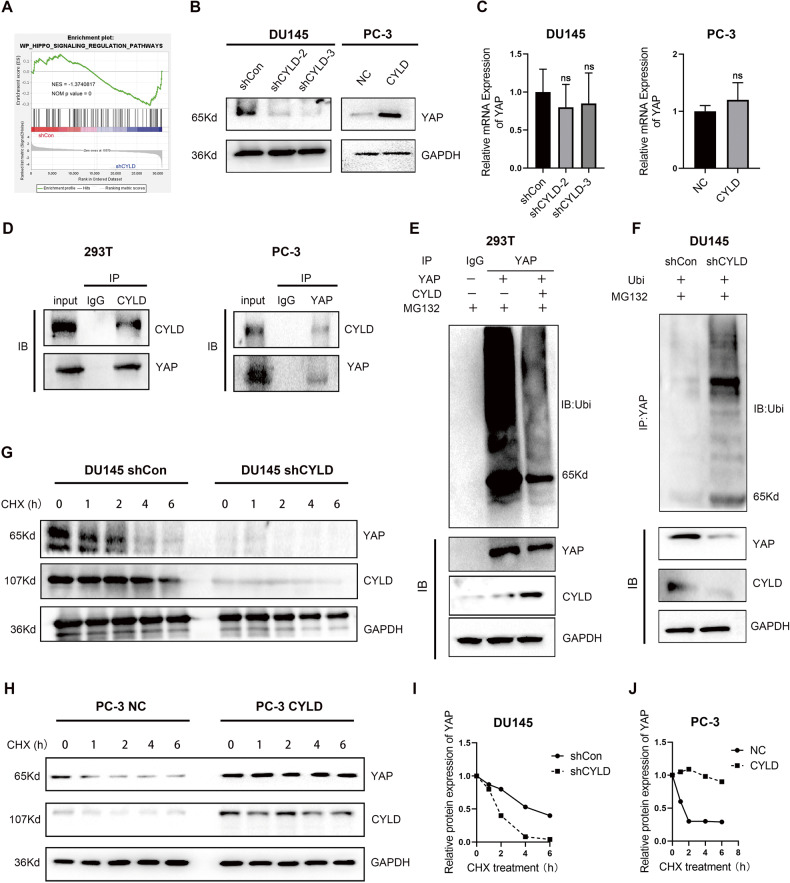
CYLD interacted with and stabilized YAP protein by deubiquitination. **A** GSEA in DU145 sublines (DU145 shCon and DU145 shCYLD) detected by RNA-seq results showing that “WP_HIPPO_SIGNALING” responded to low CYLD expression. **B** Western blot analysis of YAP expression levels in these established cell lines. **C** Quantitative real-time PCR (qRT-PCR) analysis of the mRNA expression levels of YAP in these established cell lines (*N* = 3). **D** Co-immunoprecipitation (Co-IP) assay was applied to validate the relationship between CYLD and YAP proteins in 293 T and PC-3 cells. **E** 293 T cells transfected with YAP and Ubi were pretreated with MG132 (20 μM) for 6 h, the ubiquitination assay was performed with immunoprecipitation by YAP antibody and analyzed by Western blot. **F** DU145 sublines were pretreated with MG132 (20 μM) and Ubi for 6 h, the ubiquitination assay was performed with immunoprecipitation by YAP antibody and analyzed by Western blot. **G**, **H** DU145 and PC-3 sublines were treated with 100 μg/ml CHX for the indicated time. Equal amounts of WCL were immunoblotted with the indicated antibodies. GAPDH was used as the loading control. **I**, **J** The YAP protein abundance was quantified by ImageLab and plotted as indicated. GAPDH was used as the loading control.

To identify the pathways and target genes of CYLD, we detected the gene expression profiling by high throughput RNA-sequencing in DU145 sublines (DU145/shCon and DU145/shCYLD). A gene set enrichment analysis (GSEA) of the pathways revealed a close relationship between CYLD and the Hippo pathway. The “WP_HIPPO_SIGNALING” was significant enriched in PCa groups with CYLD knockdown (Fig. 4A), which revealed that CYLD inhibited the activation of Hippo signal pathway. Western blot analysis revealed that knockdown of CYLD in DU145 cells decreased the expression of YAP protein, while overexpression of CYLD in PC-3 cells upregulated the expression of YAP protein (Fig. 4B). RT-PCR was applied to detect the expression of YAP mRNA and the results showed that there was no significant change of YAP mRNA in DU145 and PC-3 sublines (Fig. 4C), indicating that CYLD should regulate the expression of YAP at the protein level as a deubiquitinating enzyme. In order to further verify this hypothesis, we used Co-IP experiment to validate the relationship between CYLD and YAP proteins, and the results confirmed that endogenous YAP protein could interact with CYLD protein in PC-3 cells. Similarly, ectopic CYLD protein still interacts with YAP protein in 293 T cells (Fig. 4D). It is generally recognized that CYLD functions as a deubiquitinating enzyme. Thus, we investigated whether CYLD affected YAP protein stability. We subjected CYLD, YAP and Ubi overexpression in 293 T cells with 20 μM protease inhibitor MG132, and used Western blot analysis to detect and quantify Ubi protein expression. The YAP protein ubiquitination level in CYLD-overexpressing 293 T cells was significantly reduced compared to NC group (Fig. 4E). Similar results were also observed in DU145 sublines (Fig. 4F). Also, CYLD knocking-down DU145 sublines and CYLD-overexpressing PC-3 sublines were treated with CHX for 0,1, 2, 4 and 6 h, then Western blot analysis revealed that silencing CYLD could accelerate the degradation of YAP protein (Fig. 4G–J). Therefore, these results indicated that CYLD could interact with and stabilize YAP protein by deubiquitination.

### CYLD promoted ferroptosis in prostate cancer by activating ACSL4/TFRC

**Fig. 5:**
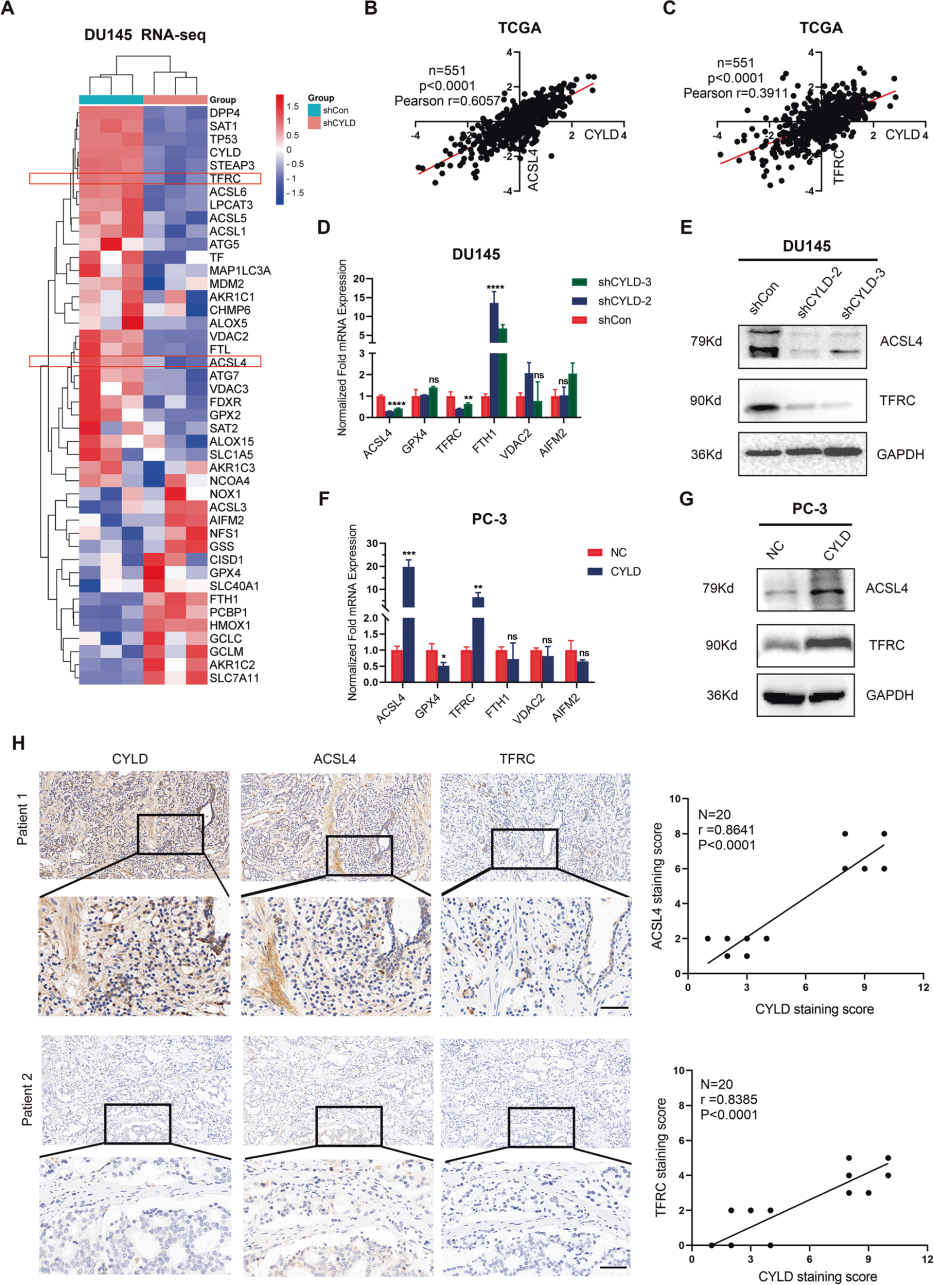
CYLD promoted ferroptosis in prostate cancer by activating ACSL4/TFRC. **A** Heatmap of ferroptosis-associated factors regulated by CYLD knockdown in DU145 sublines by RNA-seq. **B**, **C** Analysis of linear correlation between the expression level of CYLD mRNA and the expression level of ACSL4, TFRC mRNA from TCGA. **D** RT-PCR analysis of the expression of six ferroptosis-associated factors after knocking-down CYLD in DU145 cells. 18 S was applied as the endogenous control (*N* = 3). **E** Western blot analysis of ACSL4 and TFRC expression levels in DU145/shCon and DU145/shCYLD sublines. **F** RT-PCR analysis of the expression of six ferroptosis-associated factors in PC-3 sublines. **G** Western blot analysis of ACSL4 and TFRC expression levels in PC-3/NC and PC-3/CYLD sublines. **H** representative pictures and IHC analysis of the correlation between CYLD protein expression and ACSL4, TFRC protein expression in PCa specimens (*n* = 20). (*p < 0.05; **p < 0.01; ***p < 0.001; ****p < 0.0001).

CYLD deficiency reduced the sensitivity of PCa to ferroptosis in vitro and in vivo, and multiple ferroptosis-associated factors might be involved. So we crossed enrichment analysis of altered ferroptosis factors and Hippo signaling pathways in which ACSL4 and TFRC were simultaneously enriched (Fig. 5A). In addition, the expression of CYLD mRNA was positively correlated with the expression of ACSL4 mRNA (Pearson r = 0.6075, *P* < 0.0001) and TFRC mRNA (Pearson r = 0.3911, *P* < 0.0001) according to TCGA database (Fig. 5B, C). RT-PCR and Western blot analysis also revealed that CYLD knockdown decreased the mRNA and protein levels of ACSL4 and TFRC in DU145 cells, whereas CYLD overexpression upregulated the mRNA and protein levels of ACSL4 and TFRC in PC-3 cells (Fig. 5D–G). IHC staining of PCa specimens also revealed that CYLD protein expression was significantly positively correlated with ACSL4 or TFRC protein expression (Fig. 5H). Taken together, the preceding results suggested that CYLD regulated ferroptosis through increasing the expression of ferroptosis-associated factors ACSL4 and TFRC in PCa cells.

In addition, we have detected other ferroptosis-associated protein levels by Western blot analysis, such as GPX4, SLC7A11, FSP1 and GCH1 (supplemental Fig. 4A). These ferroptosis-associated factors were not affected by CYLD in DU145 and PC-3 sublines. Then the level of GSH was measured in DU145 and PC-3 sublines under RSL3 treatment, and the results showed that CYLD knockdown in DU145 cells increased the GSH compared to shCon group, while CYLD overexpression in PC-3 cells decreased the level of GSH compared to NC group (supplemental Fig. 4B). Furthermore, the iron and labile iron pool (LIP) level were measured in DU145 sublines, and we confirmed that CYLD upregulation of TFRC was associated with increased LIP (supplemental Fig. 4C).

### CYLD promoted ferroptosis in PCa cells by regulating Hippo/YAP pathway

**Fig. 6:**
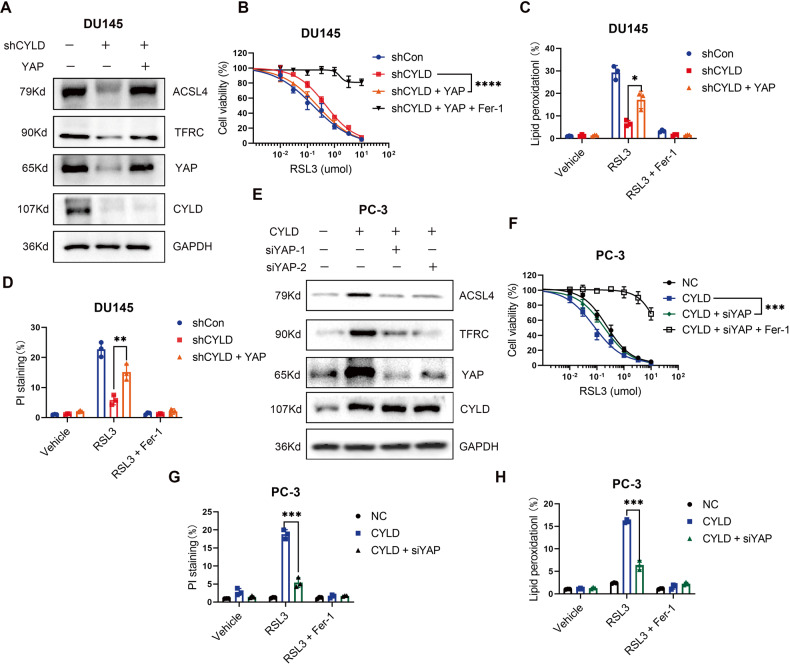
CYLD promoted ferroptosis in PCa cells by regulating Hippo/YAP pathway. **A** after overexpression of YAP in DU145/shCYLD sublines, the protein expression of ACSL4 and TFRC were examined by Western blot analysis. **B** Cell viability of DU145/shCYLD sublines after YAP overexpression and RSL3 treatment or ferrostatin-1 (Fer-1; 5 μM) treatment as indicated. **C** Lipid peroxidation measurement in DU145/shCYLD sublines after YAP overexpression and RSL3 treatment or ferrostatin-1 (Fer-1; 5 μM) for 6 h. **D** Results of PI staining of DU145/shCYLD sublines after YAP overexpression and RSL3 treatment or ferrostatin-1 (Fer-1; 5 μM) for 12 h. **E** after knocking-down YAP by siRNAs in PC-3/CYLD sublines, the protein expression of ACSL4 and TFRC were examined by Western blot analysis. **F** Cell viability of PC-3/CYLD sublines after siYAP transfection and RSL3 treatment or Ferrostatin-1 (Fer-1; 5 μM) treatment as indicated. **G**. Lipid peroxidation measurement in PC-3/CYLD sublines after siYAP transfection and RSL3 treatment for 12 h. **H** Results of PI staining of PC-3/CYLD sublines after siYAP transfection and RSL3 treatment for 20 h. (**p < 0.01; ***p < 0.001; ****p < 0.0001).

Since YAP may directly target and regulate several important ferroptosis-associated factors in epithelial cells, we further investigated whether CYLD regulated ACSL4 and TFRC expression through Hippo/YAP signals. We replenished YAP in CYLD knocking-down DU145 cells, indeed, the ACSL4 and TFRC expression levels increased after YAP rescued (Fig. 6A). Correspondingly, we detected cell viability by MTT assay and measured cell death and intracellular lipid peroxidation by flow cytometry. Whereas CYLD knockdown decreased RSL3-induced cell ferroptosis and lipid peroxidation in PCa cells, YAP overexpression rescued these changes (Fig. 6B–D). Similarly, knocking down YAP by siRNA in CYLD overexpressing PC-3 cells could suppress the expression levels of ACSL4 and TFRC (Fig. 6E). Consistently, YAP knockdown could significantly decrease cell death and lipid peroxidation levels induced by CYLD overexpression in PC-3 cells (Fig. 6F–H). Consistently, nucleus-cytoplasm extraction and immunofluorescence staining disclosed that the expression of YAP in the nucleus were significantly reduced in CYLD knocking-down DU145 cells but increased in CYLD overexpressing PC-3 cells (supplemental Fig. 5A, B). These results supported that CYLD might increase nuclear YAP accumulation to affect Hippo signal pathway activation.

### CYLD modulated PCa tumorigenesis and ferroptosis in vivo

**Fig. 7:**
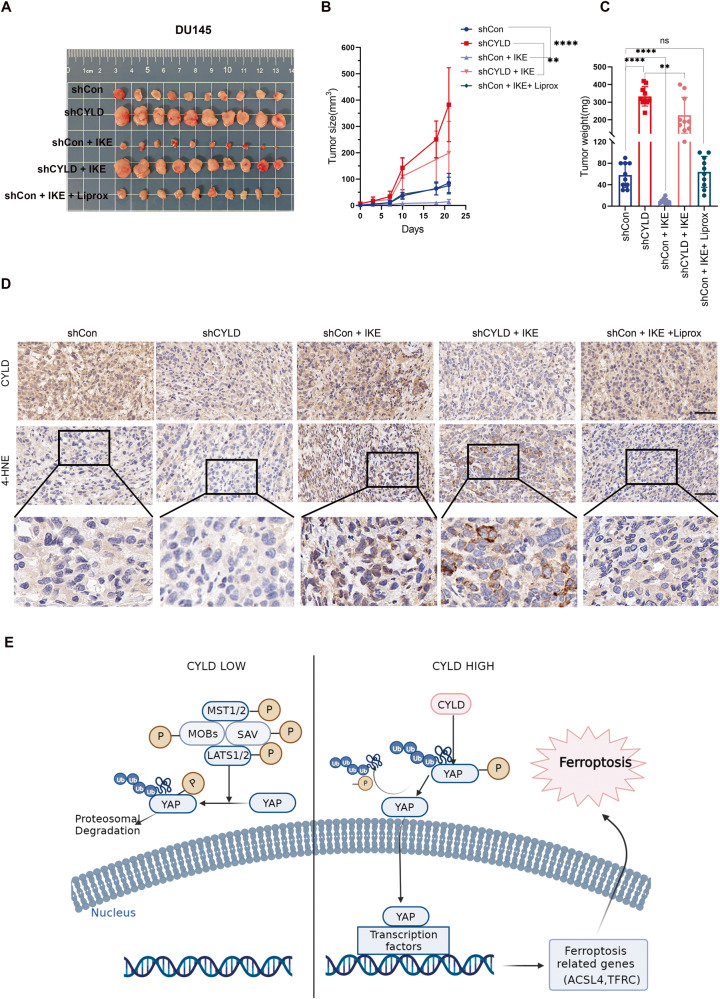
CYLD modulated PCa tumorigenesis and cell ferroptosis in vivo. **A**–**C**. Images of the tumor growth of subcutaneous xenografts established by DU145 sublines (DU145/shCon and DU145/shCYLD) after IKE treatment or Liprox treatment as indicated and the statistical results of tumor weights and volumes were shown. **D**. IHC analysis of the expression levels of CYLD and 4-HNE in xenografts. **E** a diagram showing the effects and mechanism of CYLD promoting ferroptosis in PCa through Hippo/YAP pathway (**p < 0.01; ****p < 0.0001).

We established a subcutaneous xenograft model by injecting mice with DU145 shCon and shCYLD sublines, and treating them either with the ferroptosis inducer IKE or the ferroptosis inhibitor liproxstatin-1 (Fig. 7A, and supplemental Fig. 6). We then measured all tumor volumes and wet weights every 3 d. The mice in the DU145 shCYLD group were comparatively less sensitive to IKE and the growth of their tumors was less inhibited than those in the shCon group (Fig. 7B, C). IHC analysis revealed that the xenografts in the DU145/shCon group after treatment presented a higher 4-hydroxynonenal (4-HNE) expression than those in the other groups (Fig. 7D).

## Discussion

In the past decade, there have been many new changes in the treatment of PCa, ADT is the standard therapy for the patients with metastatic PCa. However, most patients eventually develop into a lethal stage with metastatic CRPC [2, 3]. Precision medicine aims to treat advanced cancers by identifying the genes specific to them. In this study, we underlied the expression and roles of a novel deubiquitinating enzyme in PCa development and progression. We demonstrated that CYLD could stabilize the expression of YAP protein by its deubiquitination, which subsequently activated the expression of downstream ferroptosis-associated factors (i.e, ACSL4 and TFRC), then promoted ferroptosis and inhibited cell proliferation in PCa (schematic diagram as shown in Fig. 7E).

CYLD is one of of the deubiquitinase family (DUBs), which contains catalytic triad of Cys - 601, His-871 and Asp-889 domains [5, 6, 8]. CYLD is a Lys-63-specific DUB that can not hydrolyze the Lys-48 chain. Though CYLD is always considered to play as an inhibitory role in most cancer types, the type of inhibitory function is highly specific to each cancer type. In melanoma, non-small cell lung cancer and breast cancer, CYLD was shown to inhibit tumor cell migration and invasion [10, 14, 29, 30]. At present, there are fewer studies on CYLD expression and function in PCa. A recent study has shown that CYLD overexpression in PC-3 cells promoted the ubiquitination of NoxO1 protein, shortened its half-life, inhibited ROS production and modulated cell cycle [31]. Our work also confirmed that CYLD with gene mutation played an anti-oncogene role in PCa.

Ferroptosis is an adaptive process, which is crucial for eradicating carcinogenic cells. Due to different metabolism states, the sensitivity of different types of cancer cells to ferroptosis is also significantly different. Recent studies showed that PCa cells lacking retinoblastoma protein 1 (RB1) were more sensitive to ferroptosis, and RB1 deletion upregulated downstream TF-encoding E2Fs, modulated ACSL4 expression, enriched ACSL4-dependent arachidonic acid phospholipid, and promoted ferroptosis [22]. Also, another study considered Erastin as new treatment of PCa to inhibit the growth and development of advanced PCa in vivo, and ferroptosis can also enhance the therapeutic efficacy of cisplatin in PCa [23]. In addition, studies have found that the cancer cells susceptibility to ferroptosis depended on the activity of the Hippo pathway, which increased with Hippo inhibition and YAP activation [26]. Our study found that CYLD inhibited the activation of Hippo signal pathway and stabilized YAP protein by deubiquitination. About YAP, its function in PCa is complex. Cheng et al. have found that YAP1 was mainly localized in normal prostate basal epithelial cells, and increased in PCa. However, the expression of YAP1 decreased in NEPC tissues [32]. Moreover, high levels of YAP/TAZ makes cancer cells sensitive to ferroptosis and autophagy but resistant to apoptosis [26, 27]. Therefore, targeting ferroptosis may have therapeutic efficacy in metastatic PCa with YAP/TAZ activation.

ACSL4 is an important isoenzyme in the metabolism of polyunsaturated fatty acids (PUFA), which is responsible for converting coenzyme A ester into free fatty acids. The formation of acyl-Coenzyme A can activate the corresponding fatty acids for lipid peroxidation, making cells more sensitive to iron death [33]. TFRC is able to promote the extracellular iron transfer to the cell, and silencing TFRC is able to inhibit the iron death caused by Erastin [34]. In this study, we confirmed that the expression of ACSL4 and TFRC not GPX4, SLC7A11, FSP1 and GCH1 could be upregulated by CYLD, and then lead to the changes of GSH and LIP levels associated with PCa ferroptosis.

The present work showed that PCa tissues had lower CYLD mRNA and protein expression levels than normal prostate tissues, also, CYLD gene deletion mutation was common in these PCa tissues. A series of functional experiments in vitro and in vivo demonstrated that CYLD played an anti-oncogene role in PCa and CYLD might inhibit PCa progression through ferroptosis. Our study showed that CYLD stabilized the protein expression of YAP by its deubiquitination, and activated the expression of downstream ferroptosis-associated factors ACSL4 and TFRC, then promoted ferroptosis in PCa. Taken together, we have revealed the critical role of CYLD in PCa ferroptosis. For these reasons, CYLD could be a potential molecular target in PCa therapy.

### Reporting summary

Further information on research design is available in the Nature Research Reporting Summary linked to this article.

### Supplementary information

supplemental fig

Original Data File

Reporting Summary

## Data Availability

All primary data are available upon request.
