# Supplementary material for: CYLD regulates cell ferroptosis through Hippo/YAP signaling in prostate cancer progression
Source: Cell Death Dis. 2024 Jan 22;15(1):79. doi: 10.1038/s41419-024-06464-5 (PMC10800345; doi:10.1038/s41419-024-06464-5)

Supplemental Fig. 1 Expression and mutation of CYLD in PCa tissues. A. CYLD mRNA expression in PCa and their paired adjacent normal tissues from GEO databases (GSE62293 and GSE8511). **B.** CYLD deletion in PCa tissues by analyzing data mined from cBioPortal. **C.** The gene sequencing reports of PCa patients from eight different Chinese hospitals. Of these cases, 421 patients presented CYLD mutations. The principal nucleic acid mutations and the main amino acid mutation were showed (* $p < 0.05$; **** $p < 0.0001$).

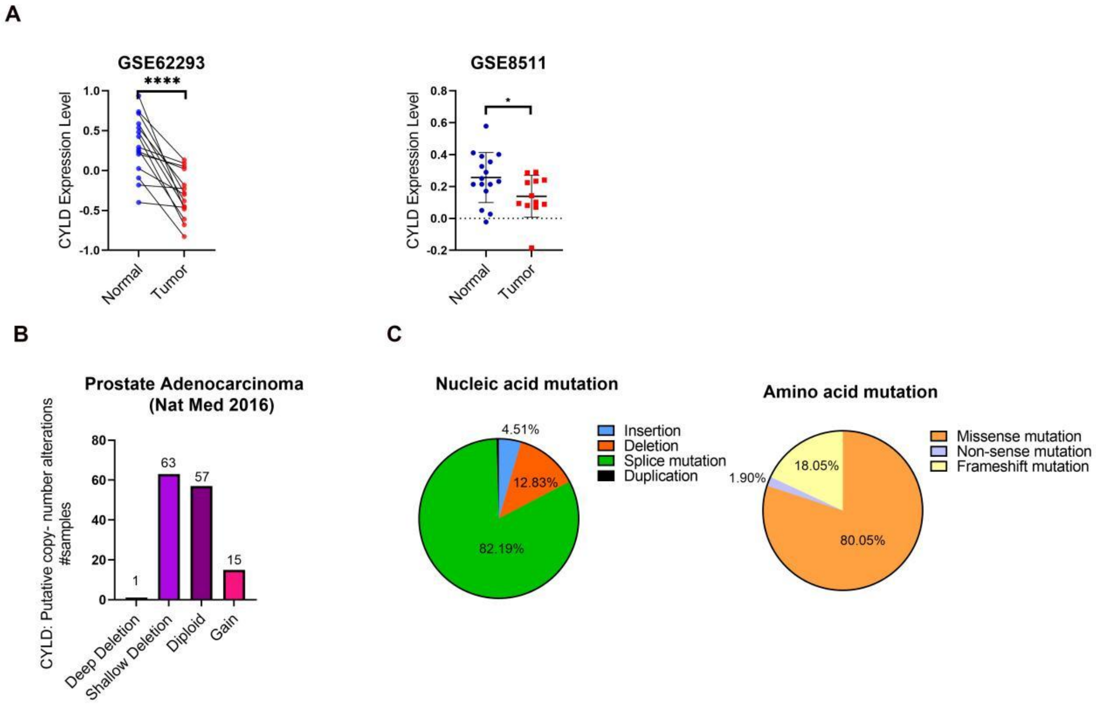

Supplemental Fig. 2 CYLD impeded malignant PCa progression *in vitro.* A. Colony formation assays of 22RV1/CYLD sublines and quantitative analysis was shown (N = 3). **B.** Colony formation assays of PC-3/CYLD sbulines and quantitative analysis was shown (N = 3). **C.** Western blot analysis of CYLD expression in DU145 cell lines transfected with CYLD lentivirus and negative control. **D.** Cell viability was detected by MTT assay in DU145 transfected with CYLD lentivirus and negative control ($**p < 0.01$， $***p < 0.001$).

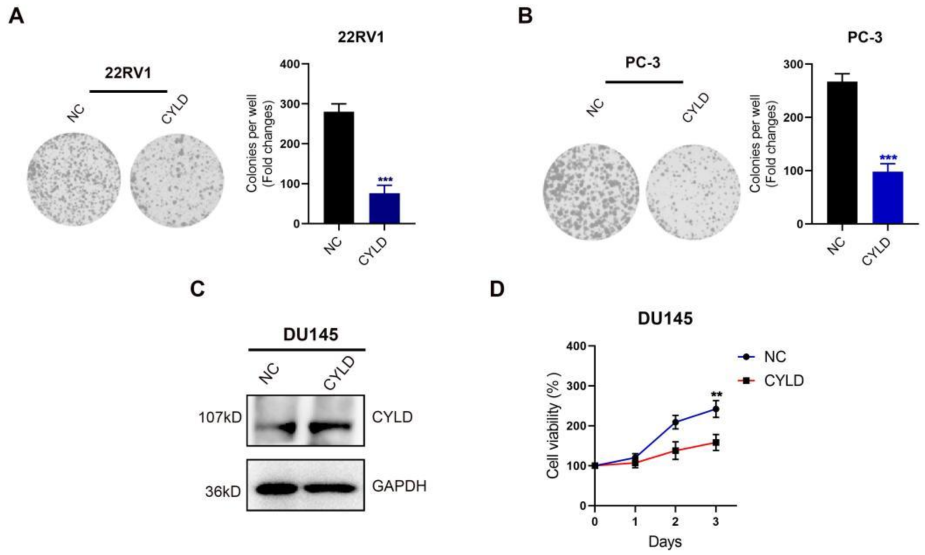

Supplemental Fig. 3 The effects of CYLD on the sensitivity of PCa cells to ferroptosis and PCa cell proliferation *in vitro*. A. Transcriptome high-throughput sequencing (HTS) and GSEA on samples of PC-3/NC and PC-3/CYLD sublines. **B.** Cell viability of DU145/CYLD and DU145/NC sublines after RSL3 treatment or ferrostatin-1 (Fer-1; 5 μM) treatment as indicated. **C.** Cell viability of DU145/CYLD and DU145/NC sublines after Erastin treatment or Ferrostatin-1 (Fer-1; 5 μM) treatment as indicated. **D.** Lipid peroxidation measurement in DU145/CYLD and DU145/NC sublines treated with RSL3 for 6 h, and quantitative analysis was shown (N = 3). **E.** Results of PI staining of DU145/CYLD and DU145/NC sublines treated with RSL3 for 12h. **F.** GSEA for RNA sequencing of PCa sublines with CYLD overexpression, and the results showed the close relationship between CYLD and cell cycle pathway. **G.** Cell cycle analysis of DU145 and PC-3 sublines using flow cytometry. **H.** Western blot analysis of cyclin D1 protein expression in these established cell lines （ $*p < 0.05$，$**p < 0.01$).

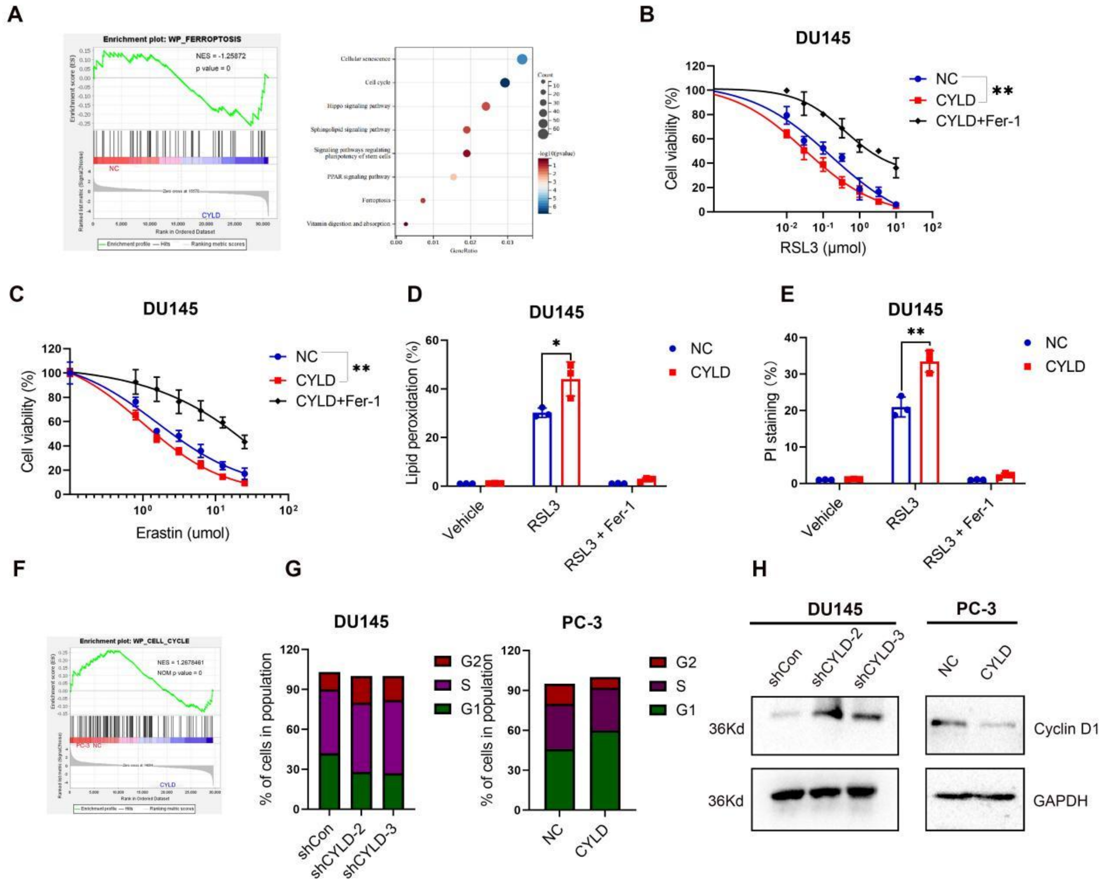

Supplemental Fig. 4 The effects of CYLD on the sensitivity of PCa cells to ferroptosis *in vitro*. A. Western blot analysis of other ferroptosis protein expression in these established cell lines. **B.** Total intracellular GSH levels were measured by QuantiChrom GSH assay in these established cell lines. **C.** FerroOrange-stained DU145/shCon, DU145/shCYLD and DU145/CYLD cells were subjected to flow cytometry analyses and images were captured ($*p < 0.05$， $**p < 0.01$).

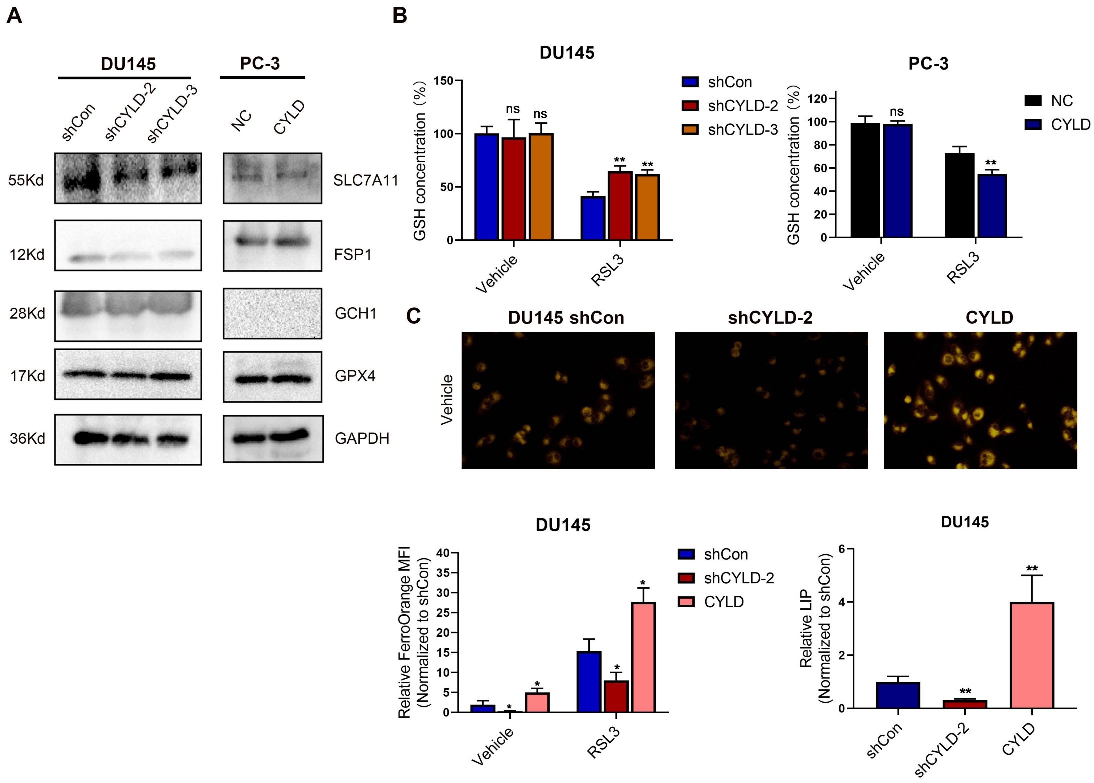

Supplemental Fig. 5 CYLD changed the nuclear location of YAP in PCa cells. A. The protein expression level of YAP in nuclear and cytosolic fractions was analyzed by Western blot in established cell lines. Lamin B1 and GAPDH were used as the loading controls, respectively. **B.** The distribution of YAP was showed by immunofluorescence, and the staining of YAP was green in DU145 established cell lines and it was red in PC-3 established cell lines. DAPI was shown blue.

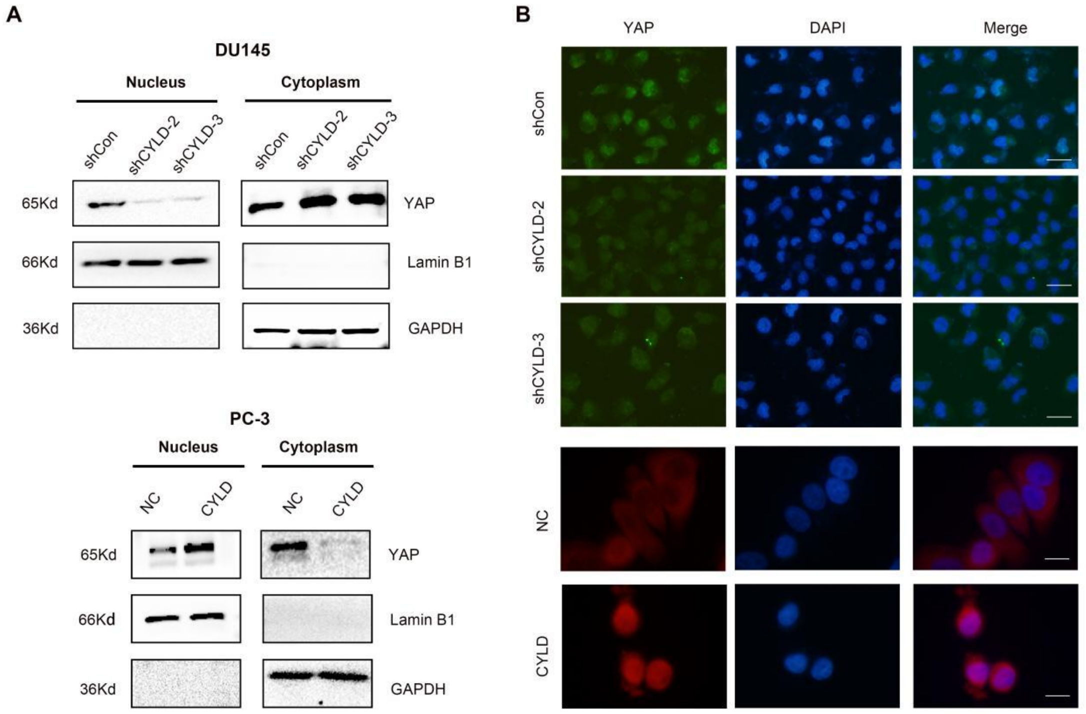

Supplemental Fig. 6 CYLD promotes ferroptosis *in vivo*. Left panel, Flow chart of treatment of the subcutaneously xenograft model with ferroptosis inducer or inhibitor in nude mice, and all tumor weights were measured every 3 d. Right panel, these treatments of the subcutaneously xenograft model had no effects on mice weight.

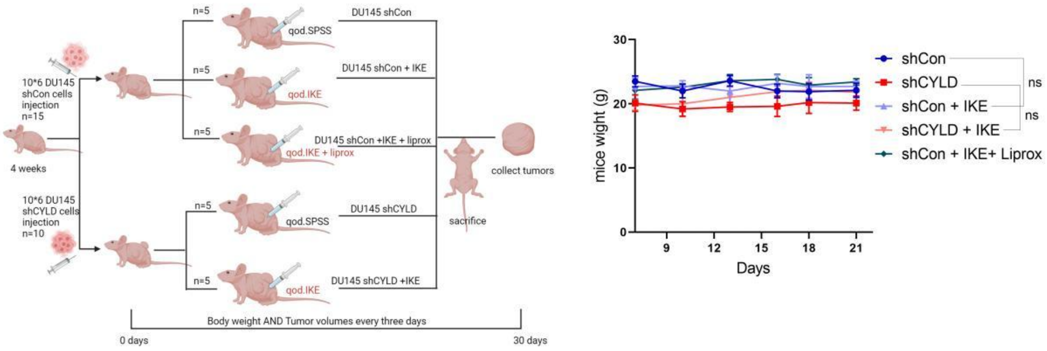

Supplement: Supplementary file 1 — supplemental fig [file 41419_2024_6464_MOESM1_ESM.pdf]
