# Supplementary material for: CYLD regulates cell ferroptosis through Hippo/YAP signaling in prostate cancer progression
Source: Cell Death Dis. 2024 Jan 22;15(1):79. doi: 10.1038/s41419-024-06464-5 (PMC10800345; doi:10.1038/s41419-024-06464-5)
Supplement: Supplementary file 2 — Original Data File [file 41419_2024_6464_MOESM2_ESM.pdf]

fig2A-CYLD：

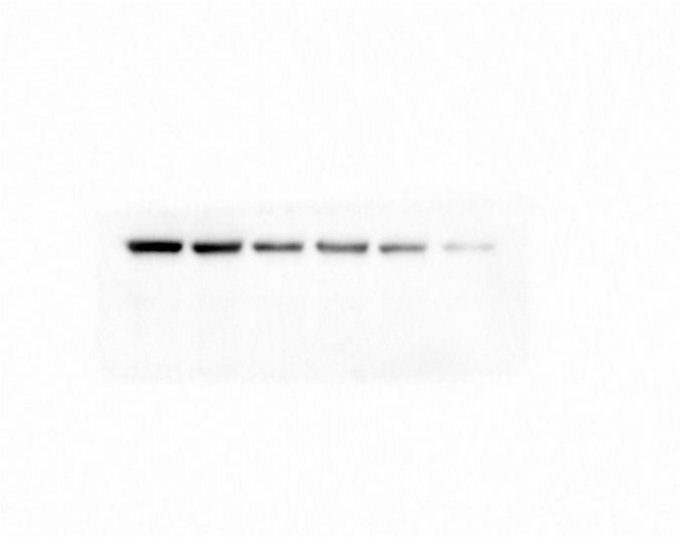

fig2A-GAPDH：

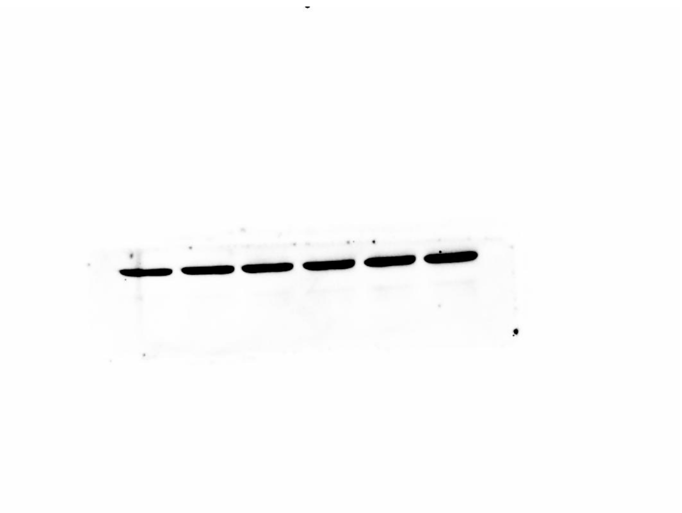

fig2C-CYLD-DU145：

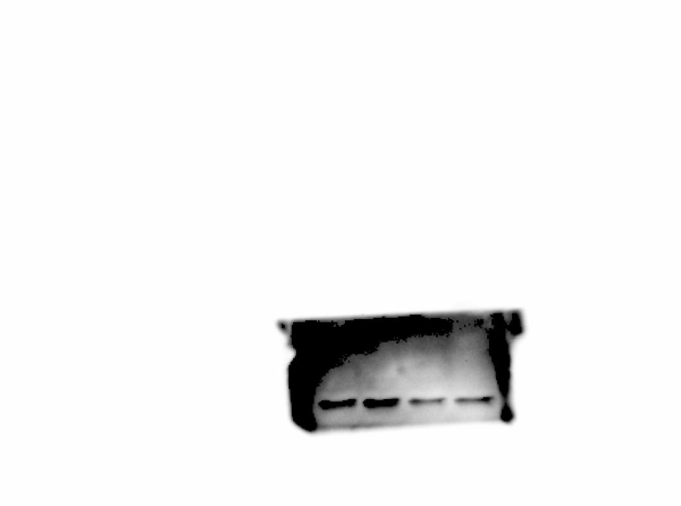

fig2C-GAPDH-DU145：

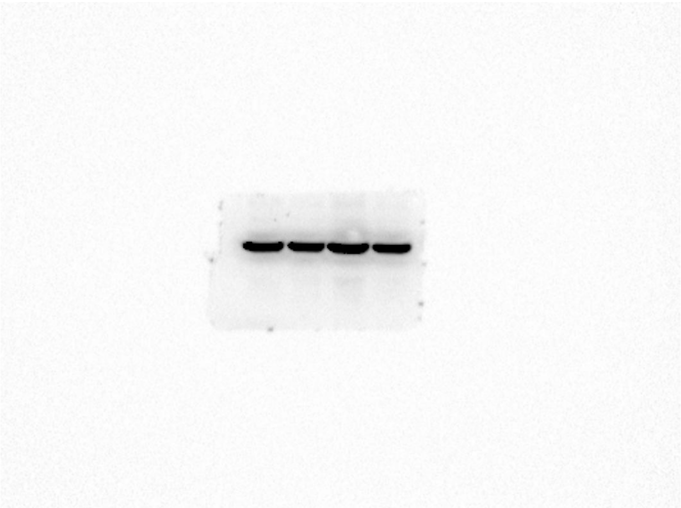

fig2D-CYLD-22RV1：

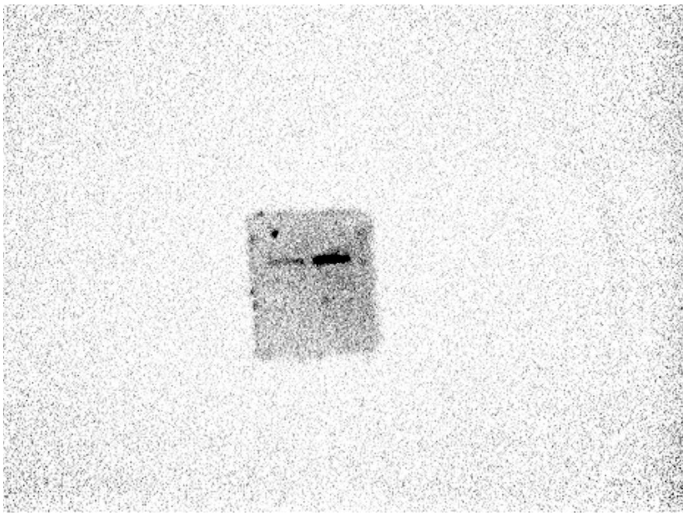

fig2D-GAPDH-22RV1：

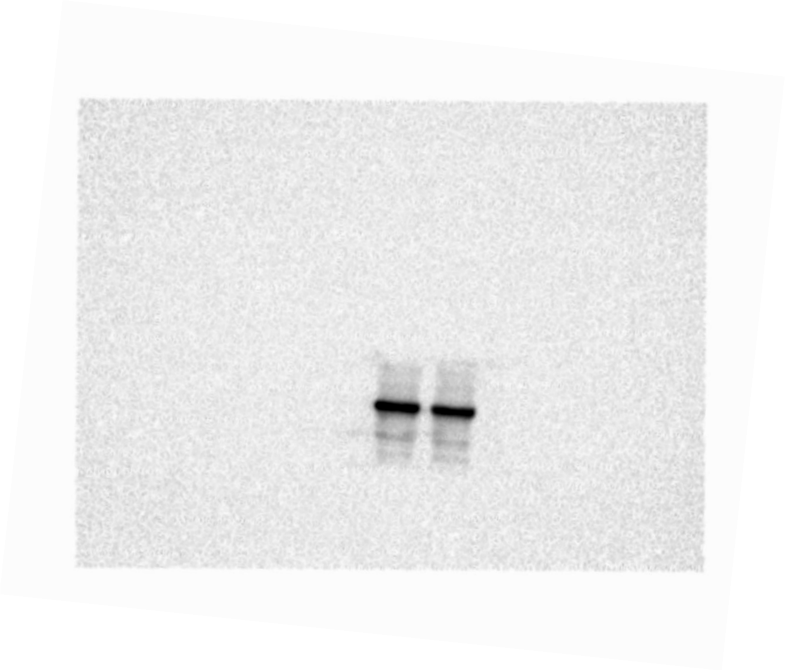

fig2D-CYLD-PC-3：

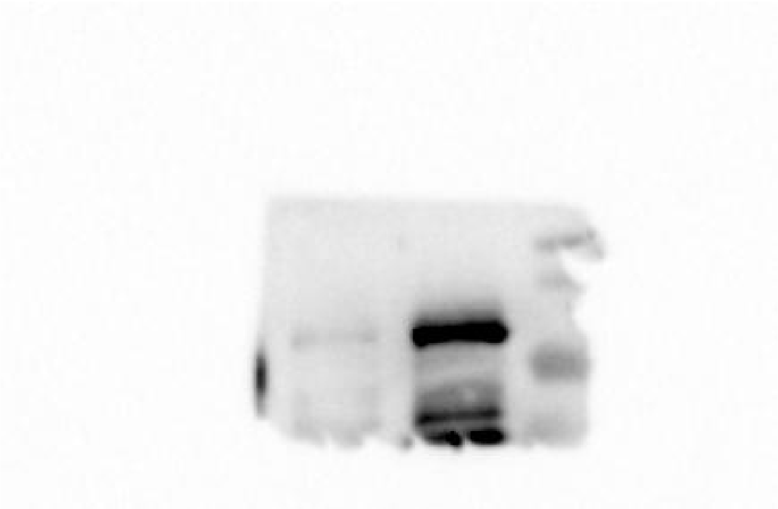

fig2D-GAPDH-PC-3：

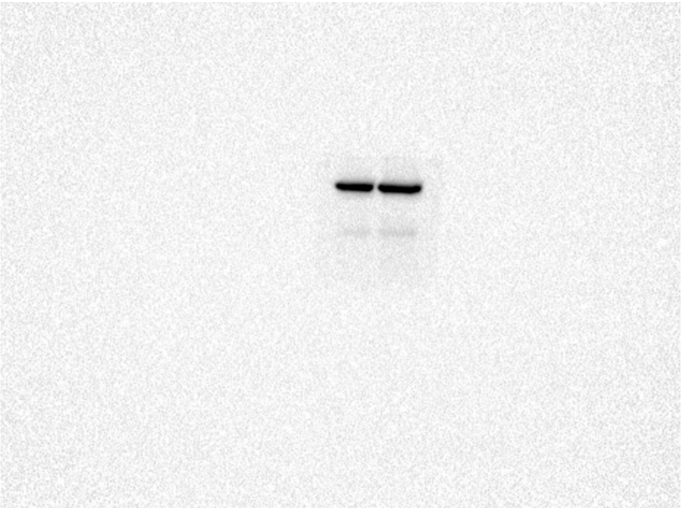

fig4B-YAP-DU145：

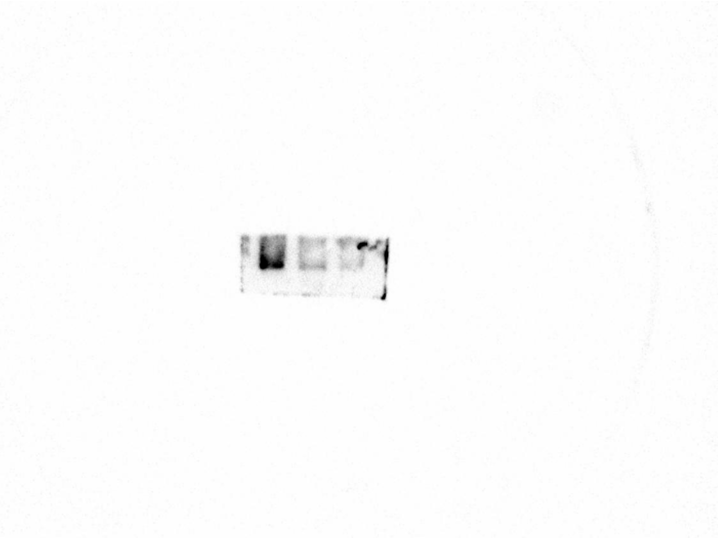

fig4B-GAPDH-DU145：

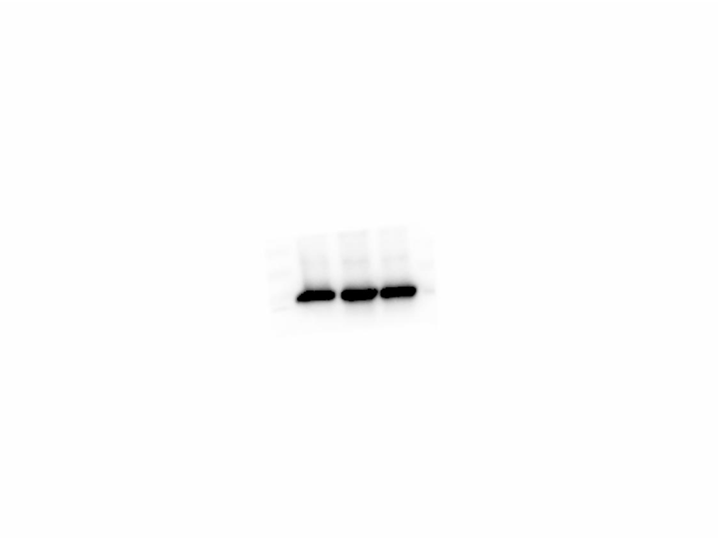

fig4B-YAP-PC-3：

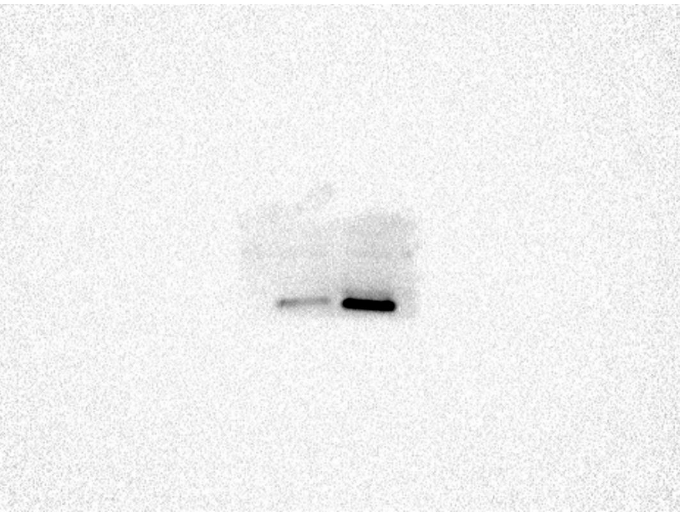

fig4B-GAPDH-PC-3：

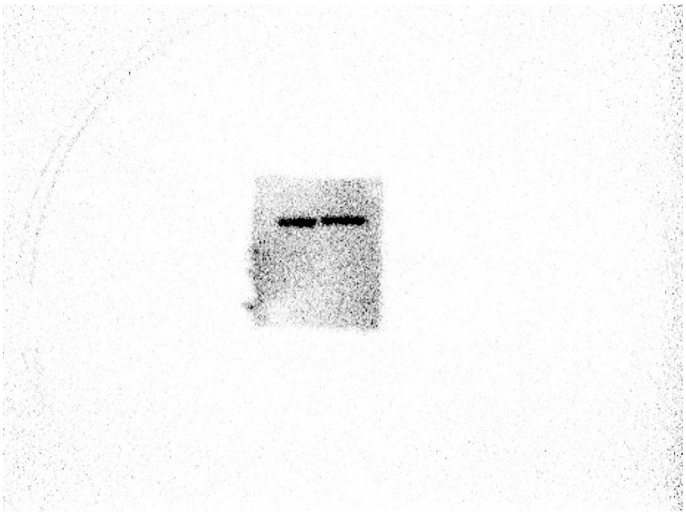

fig4D-CYLD-293T：

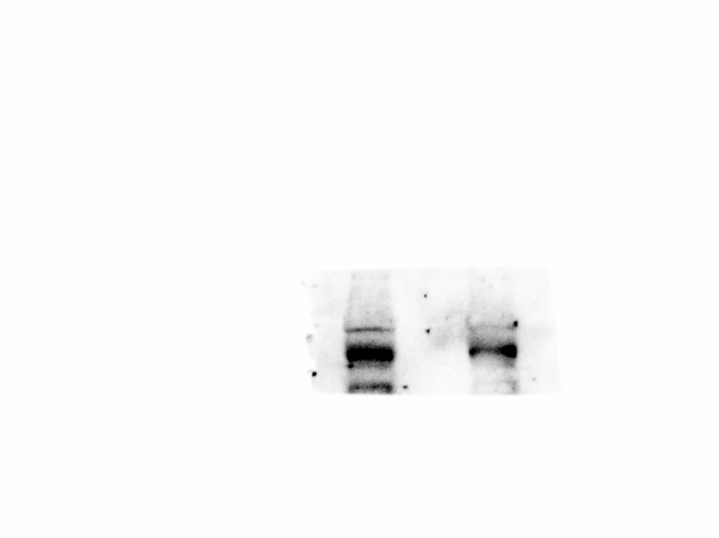

fig4D-YAP-293T：

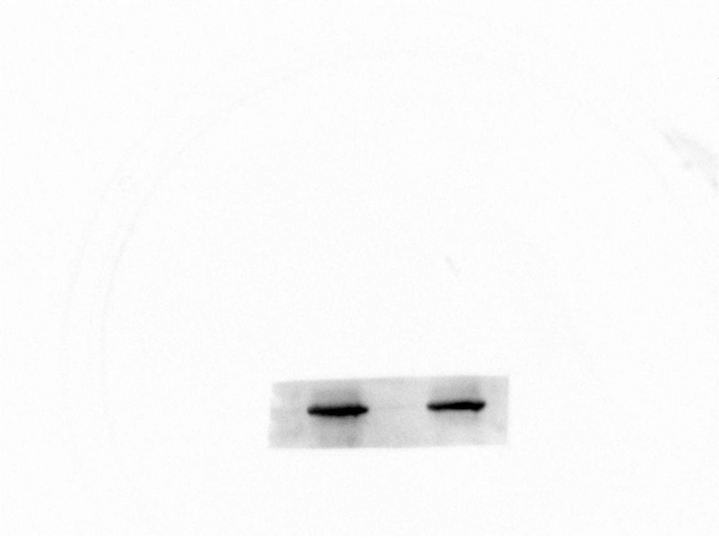

fig4D-CYLD-PC-3：

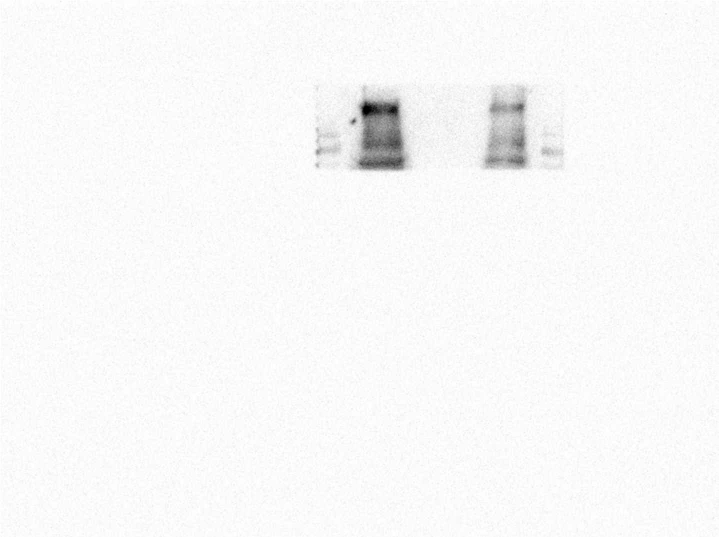

fig4D-YAP-PC-3：

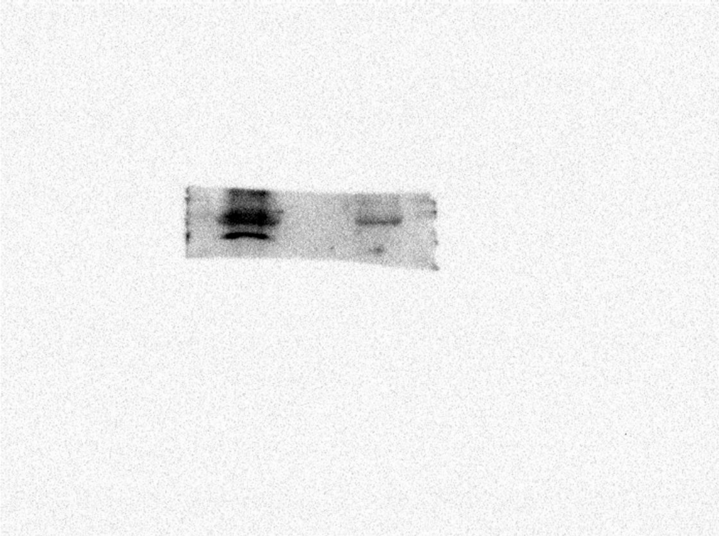

fig4E-Ubi-293T：

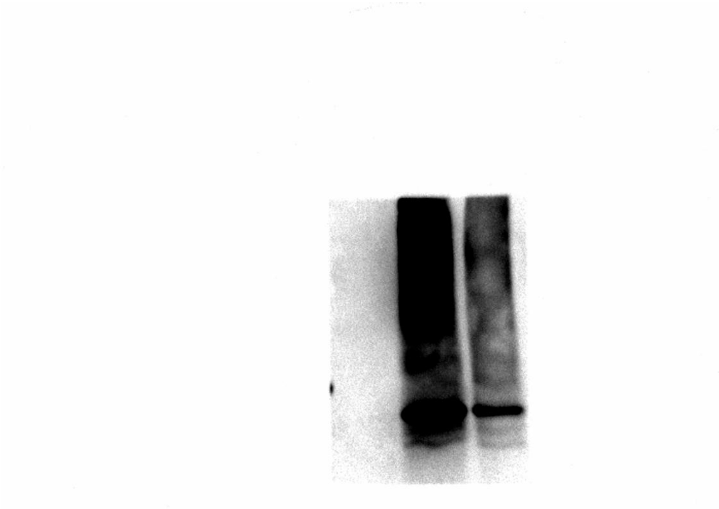

fig4E-YAP-293T：

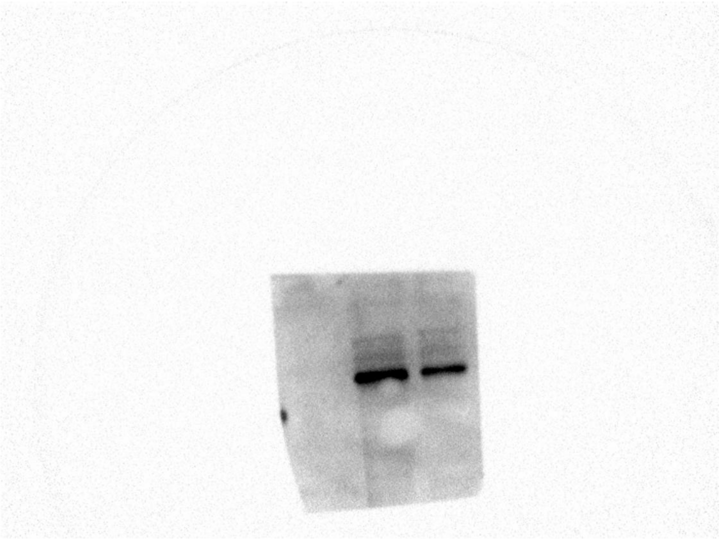

fig4E-CYLD-293T：

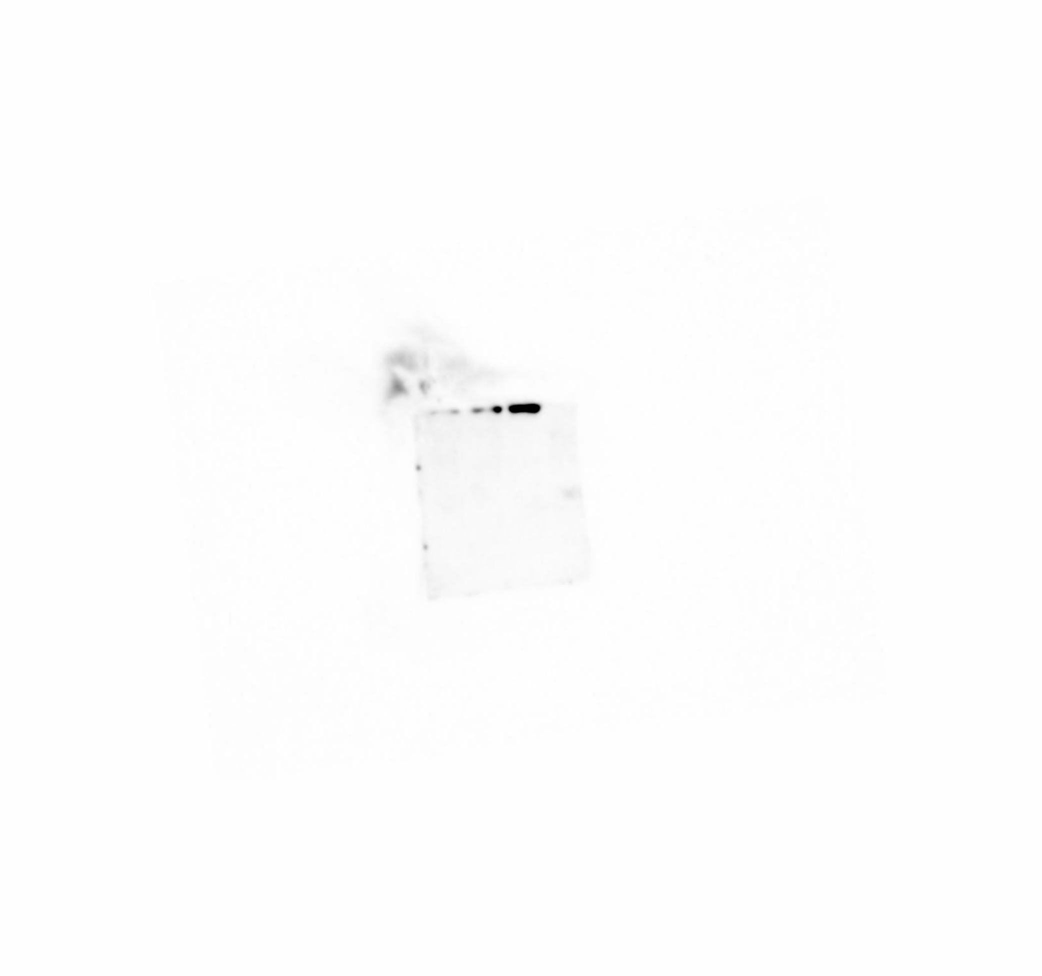

fig4E-GAPDH-293T：

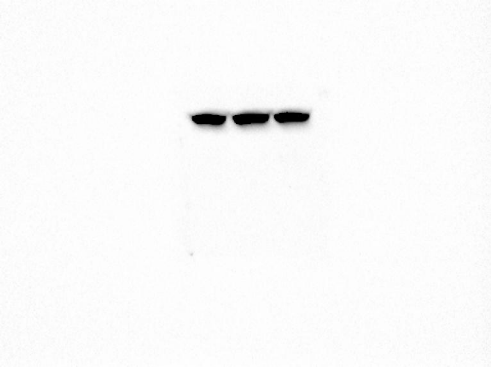

fig4F-Ubi-DU145：

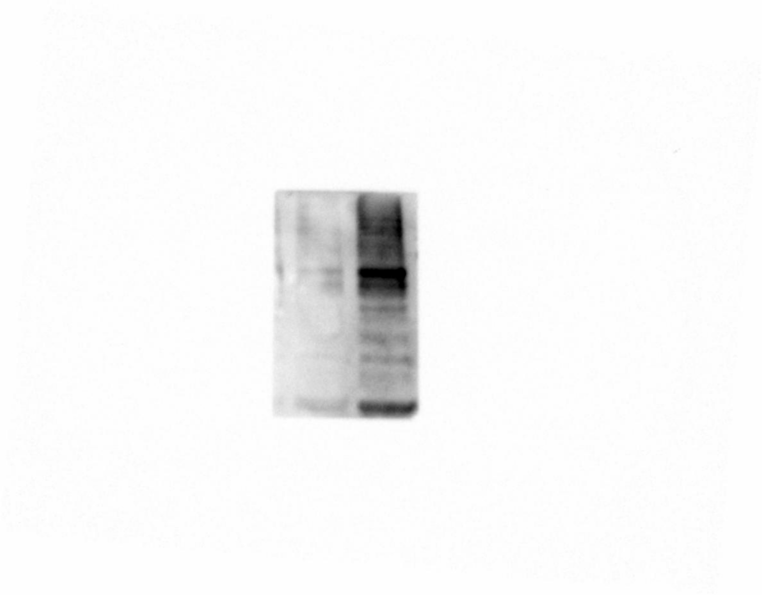

fig4F-CYLD-DU145：

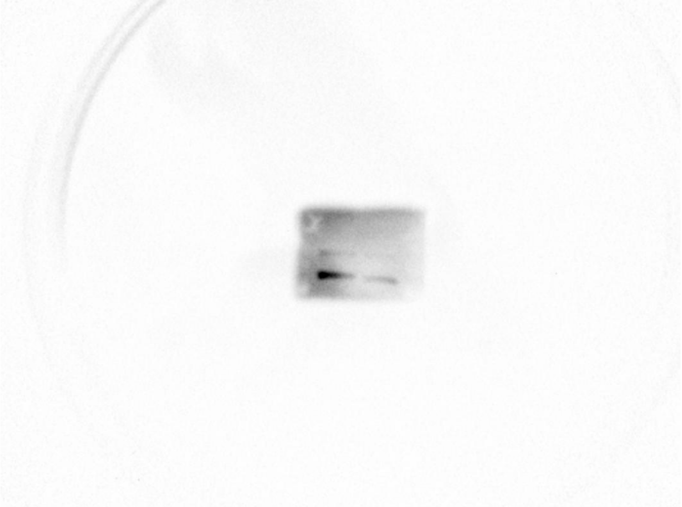

fig4F-YAP-DU145：

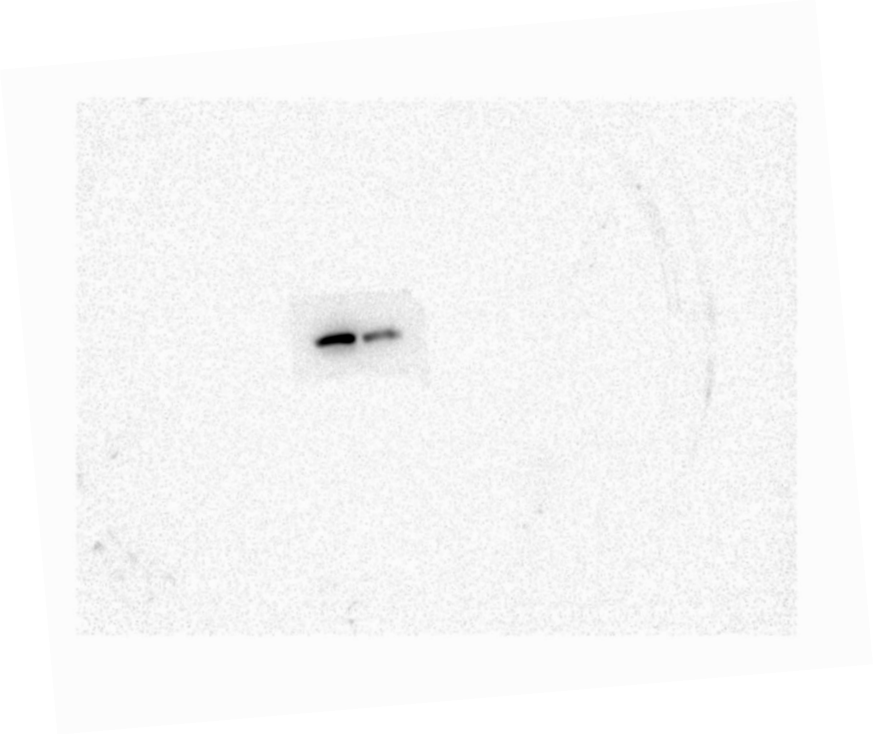

fig4F-GAPDH-DU145：

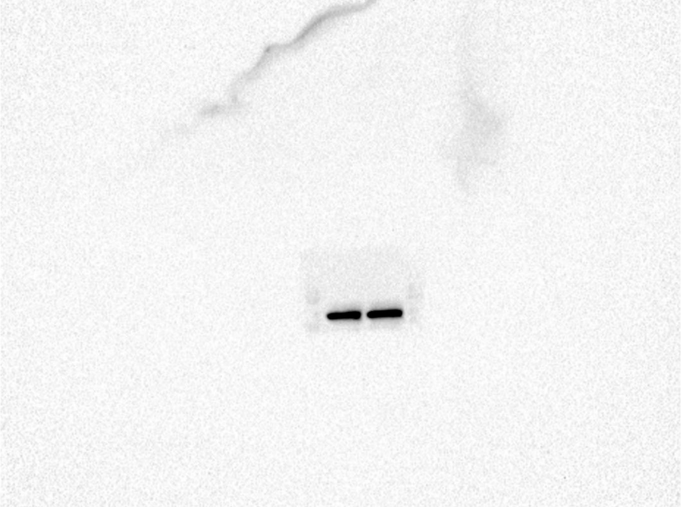

fig4G-YAP-DU145：

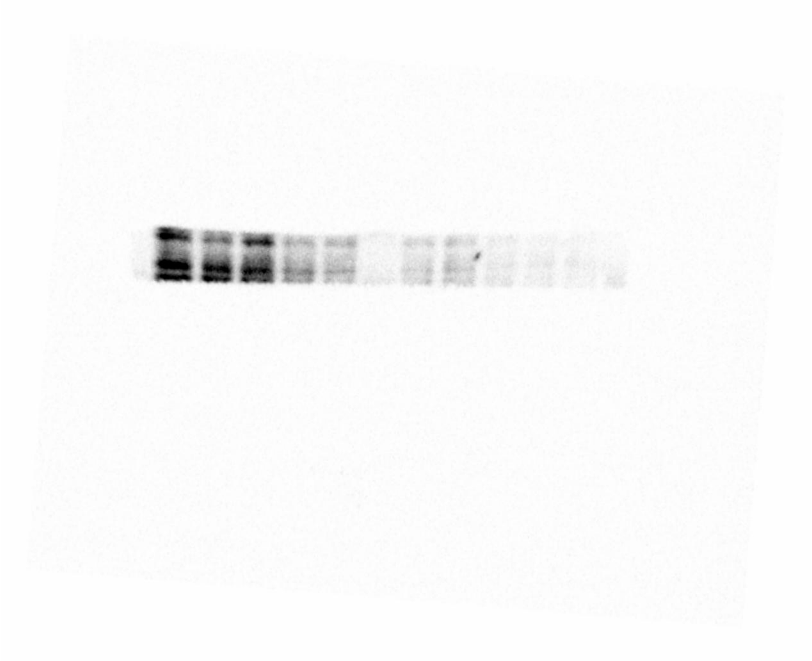

fig4G-CYLD-DU145：

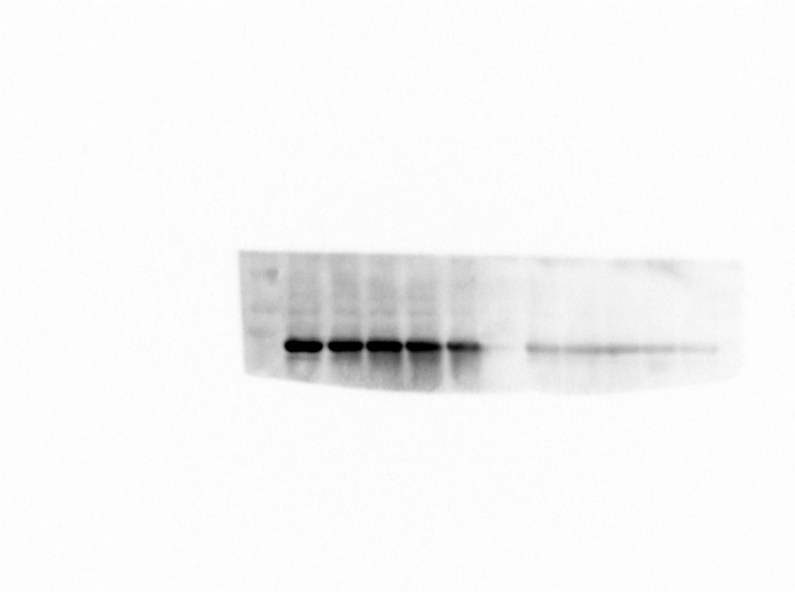

fig4G-GAPDH-DU145：

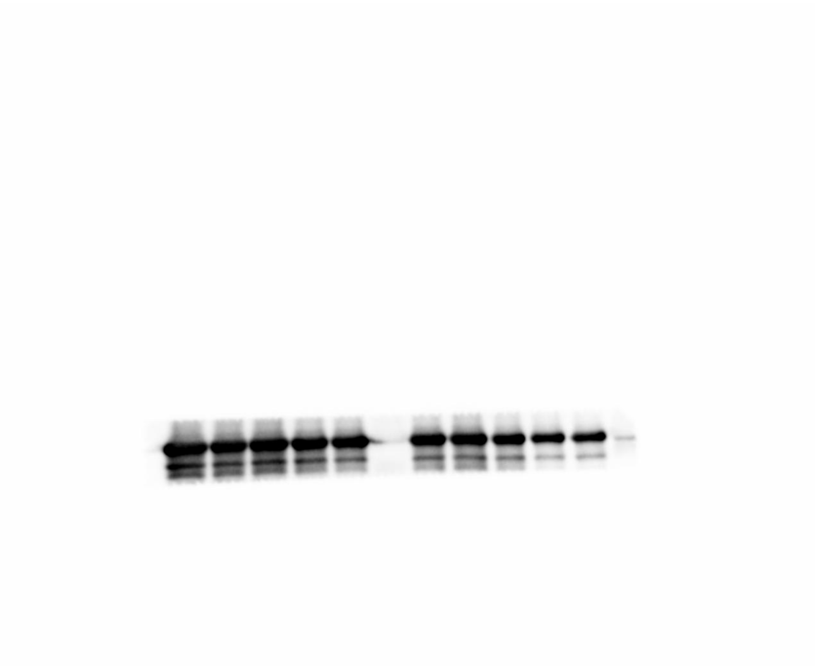

fig4H-YAP-PC-3：

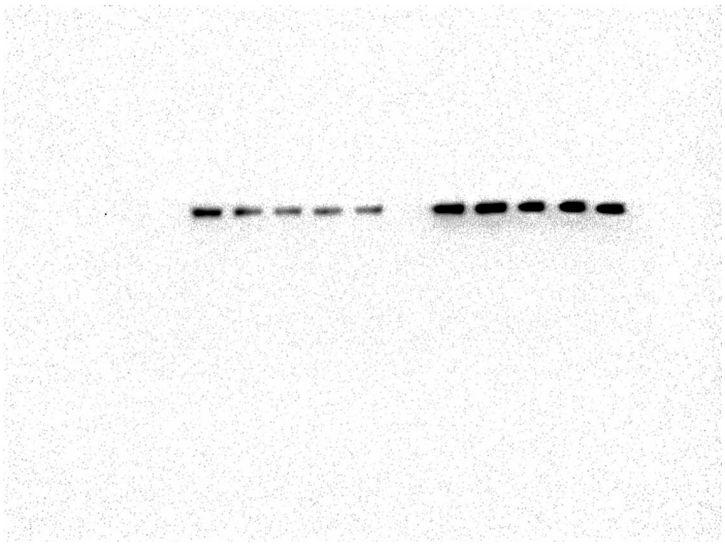

fig4H-CYLD-PC-3：

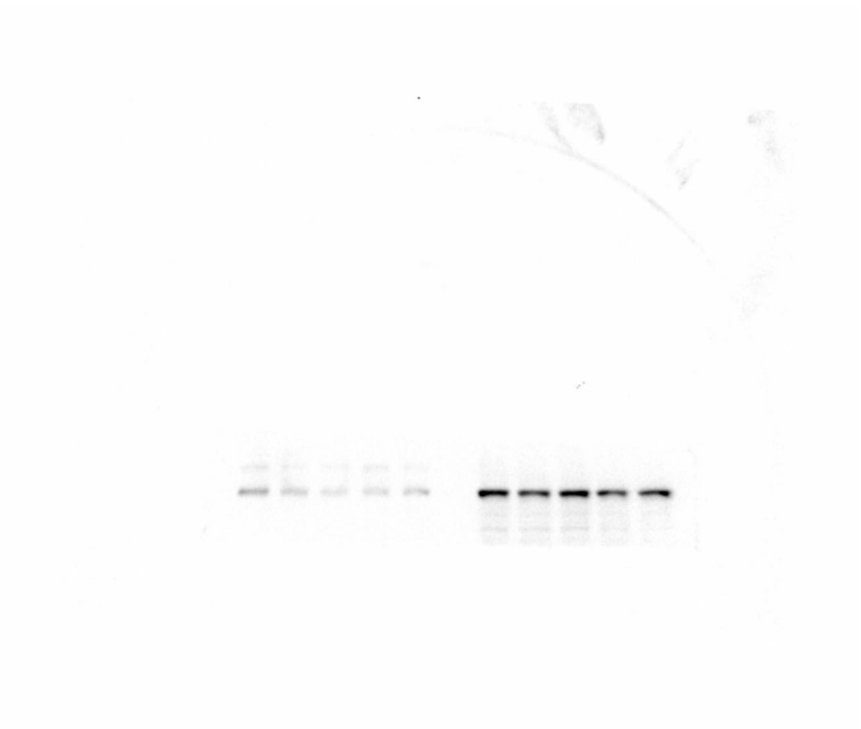

fig4H-GAPDH-PC-3：

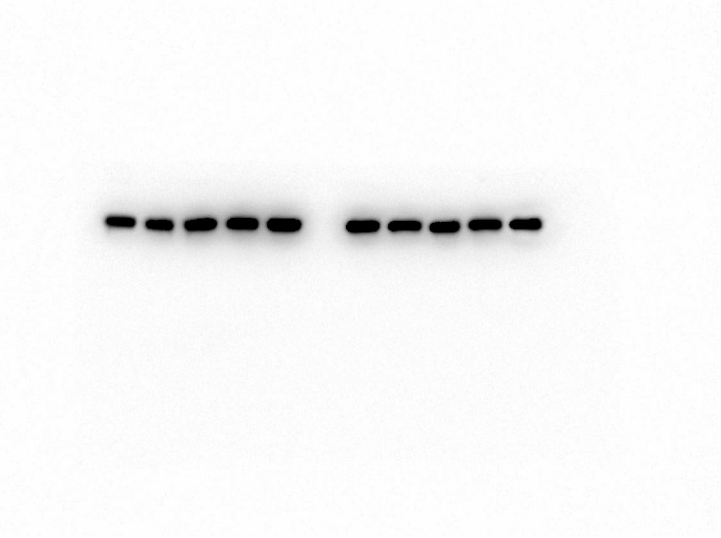

fig5E-ACSL4-DU145：

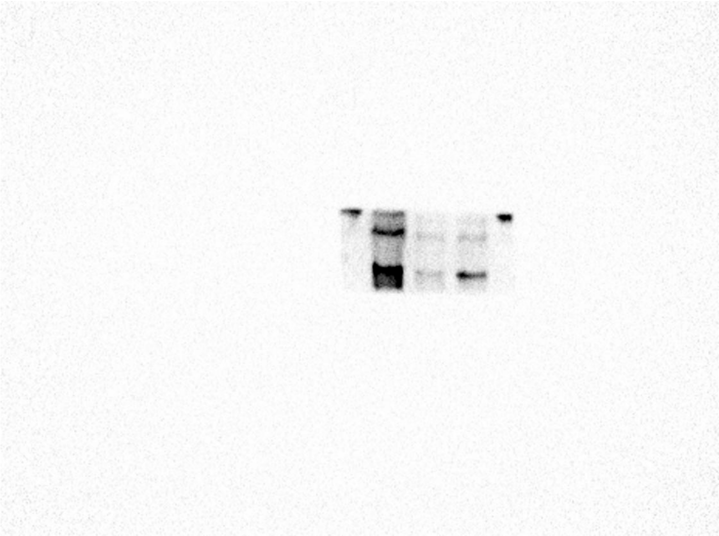

fig5E-TFRC-DU145：

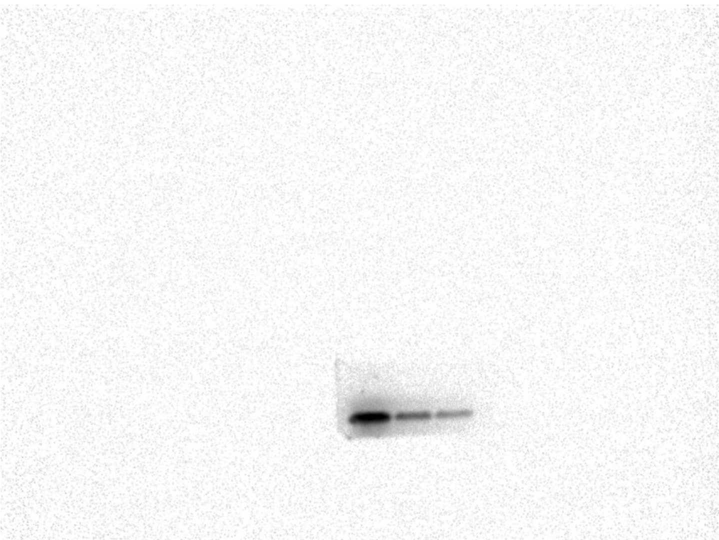

fig5E-GAPDH-DU145：

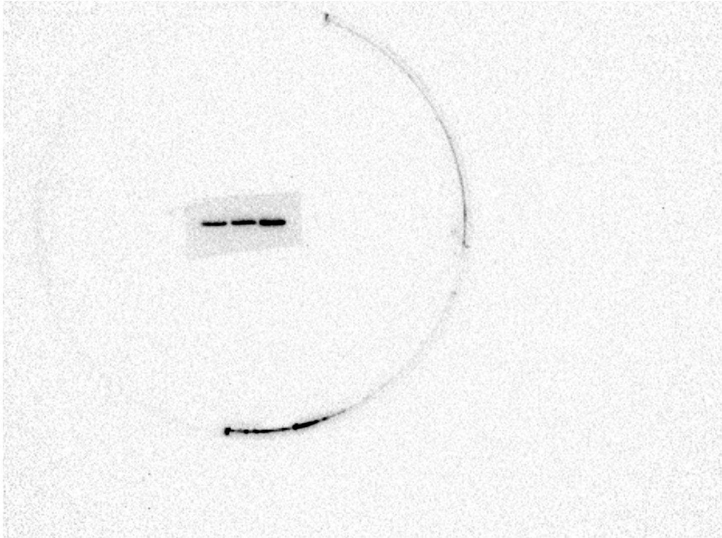

fig5G-ACSL4-PC-3：

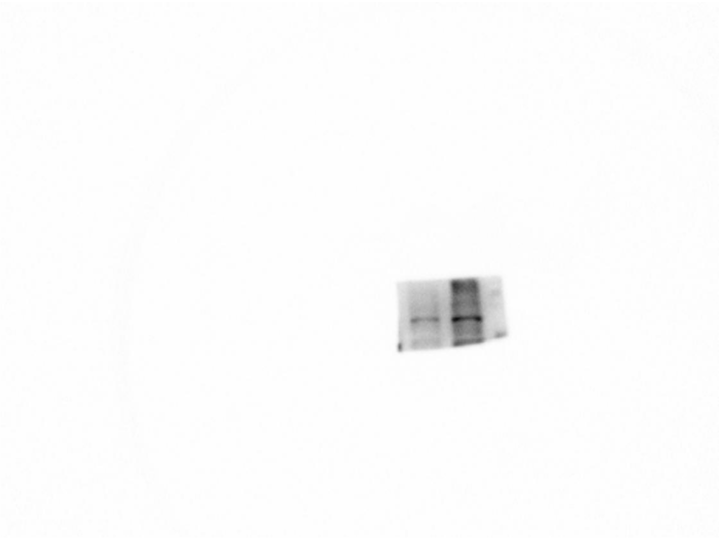

fig5G-TFRC-PC-3：

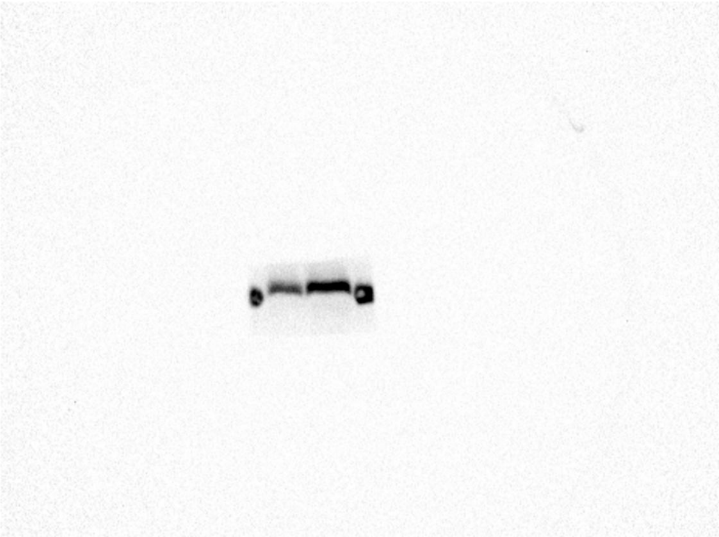

fig5G-GAPDH-PC-3：

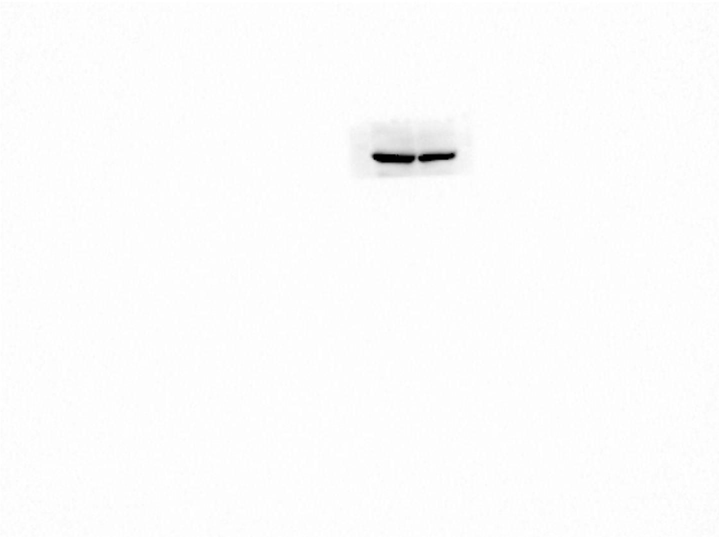

fig6A-ACSL4-DU145：

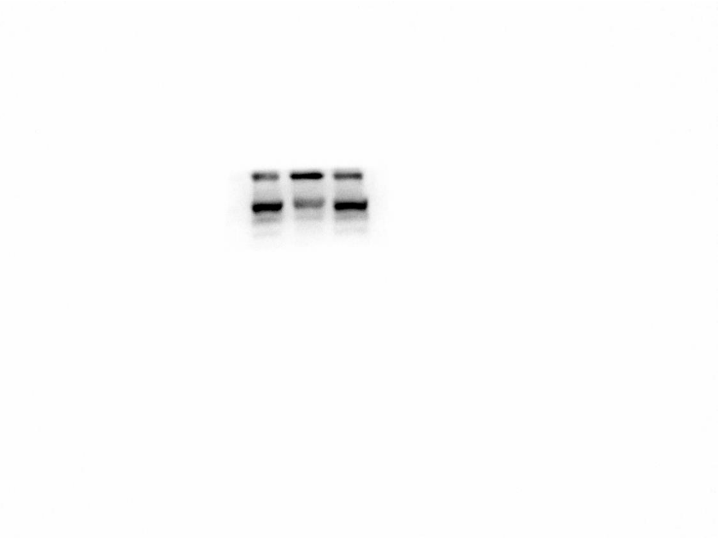

fig6A-TFRC-DU145：

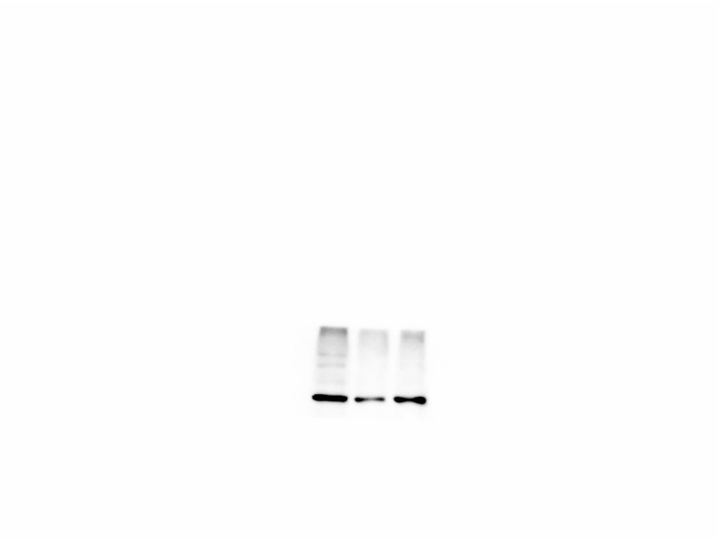

fig6A-YAP-DU145：

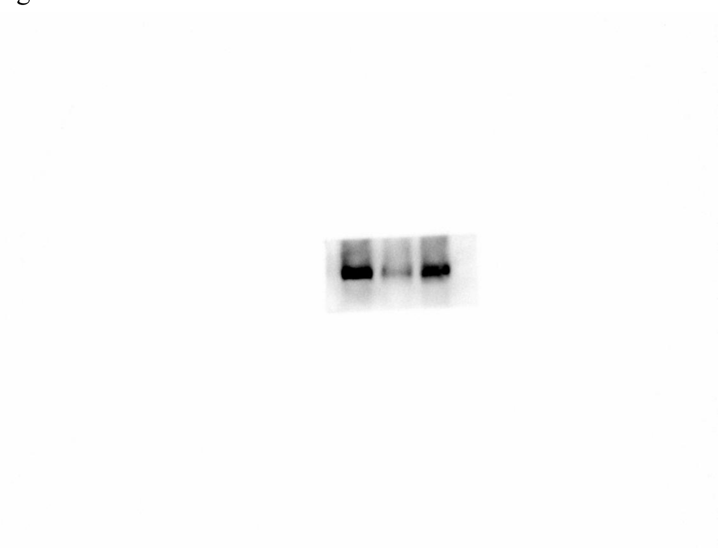

fig6A-CYLD-DU145：

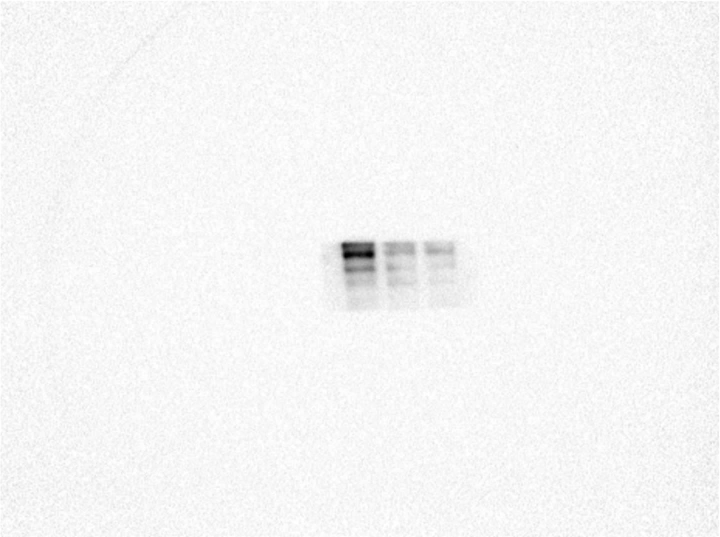

fig6A-GAPDH-DU145：

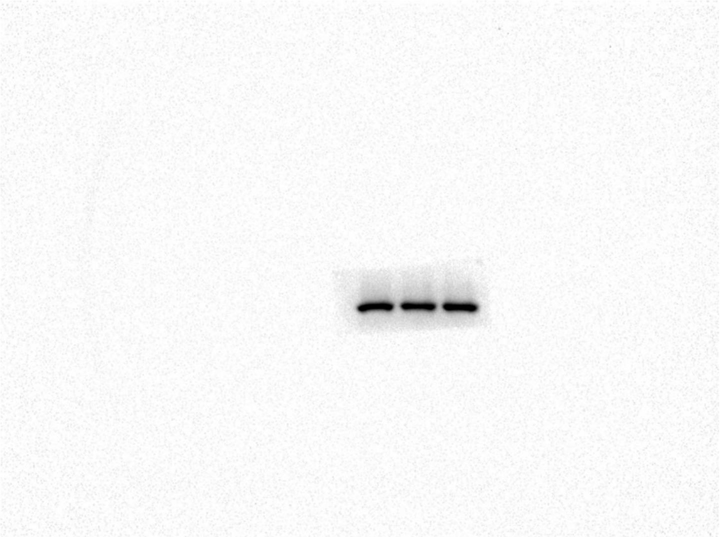

fig6E-ACSL4-PC-3：

fig6E-TFRC-PC-3：

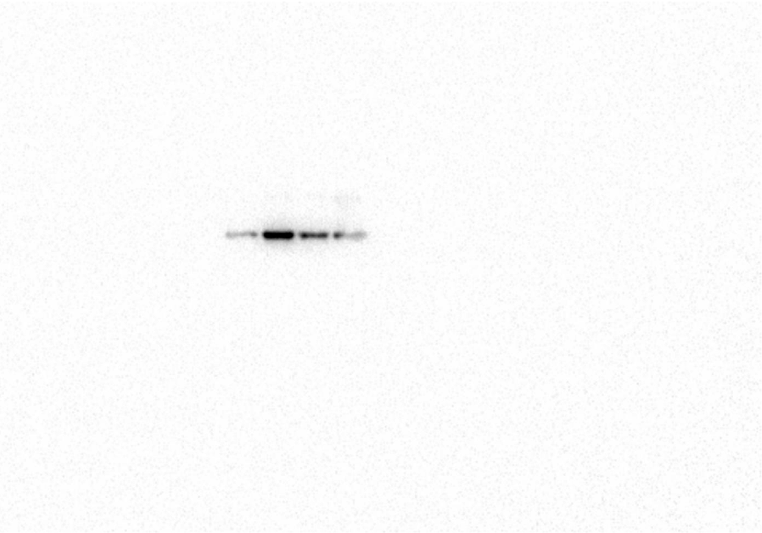

fig6E-YAP-PC-3：

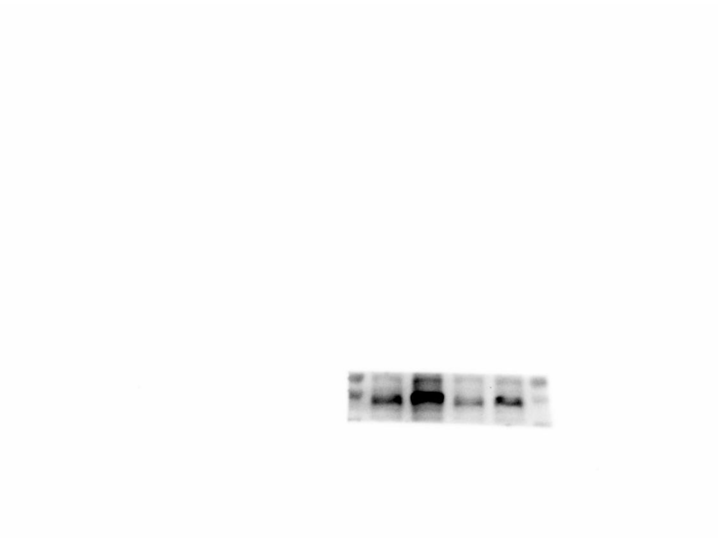

fig6E-CYLD-PC-3：

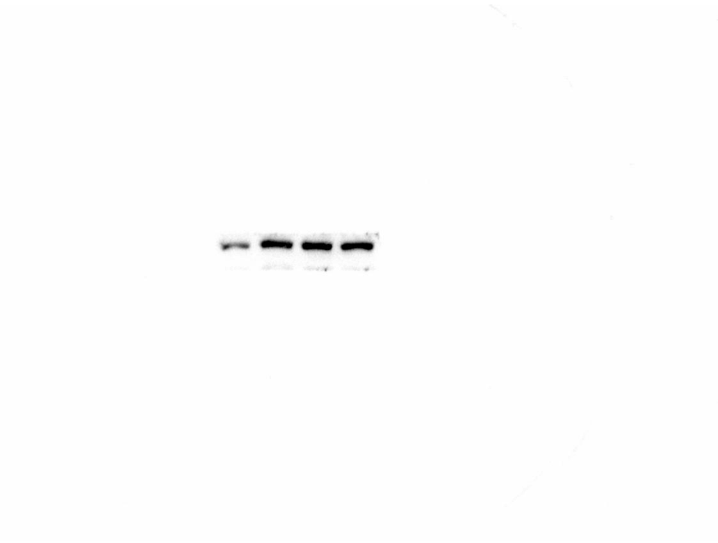

fig6E-GAPDH-PC-3:

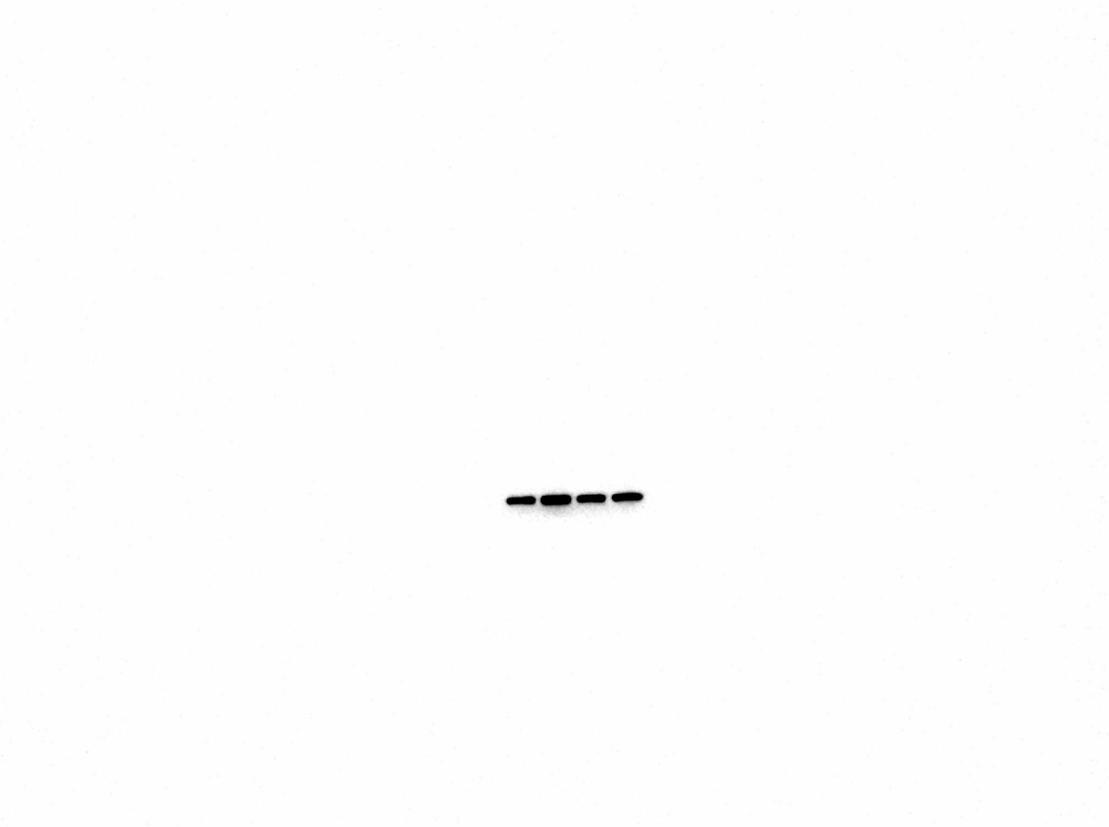

supplemental 2C-CYLD-DU145:

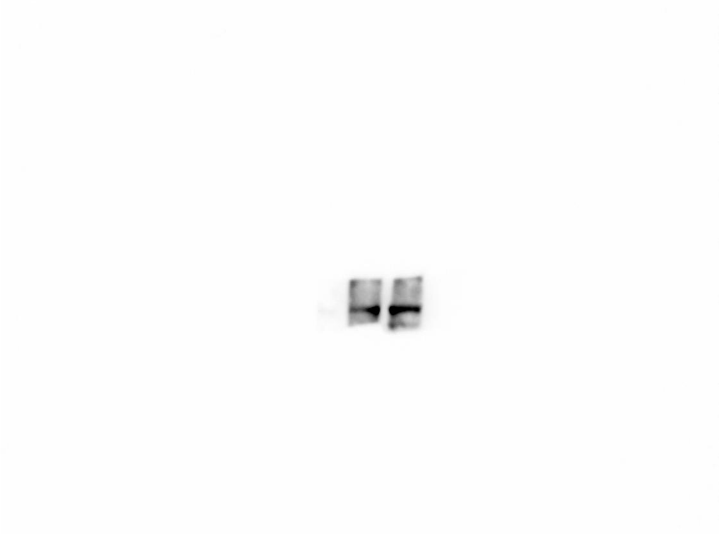

supplemental 2C-GAPDH-DU145:

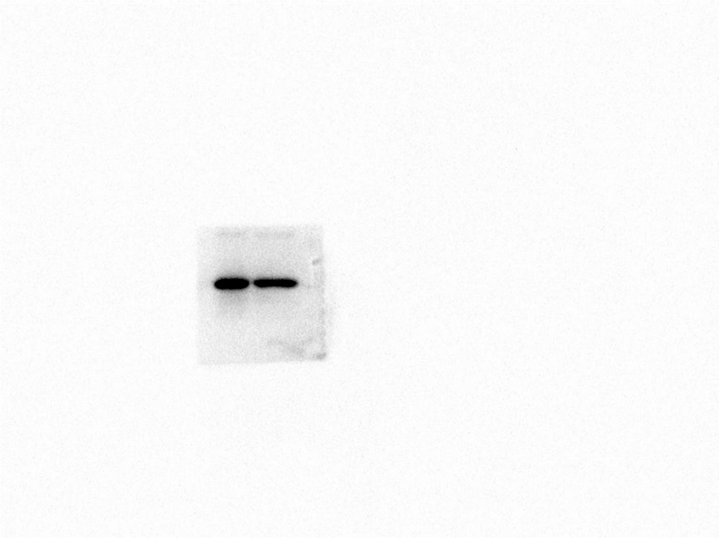

supplemental 3H-cyclinD1-DU145:

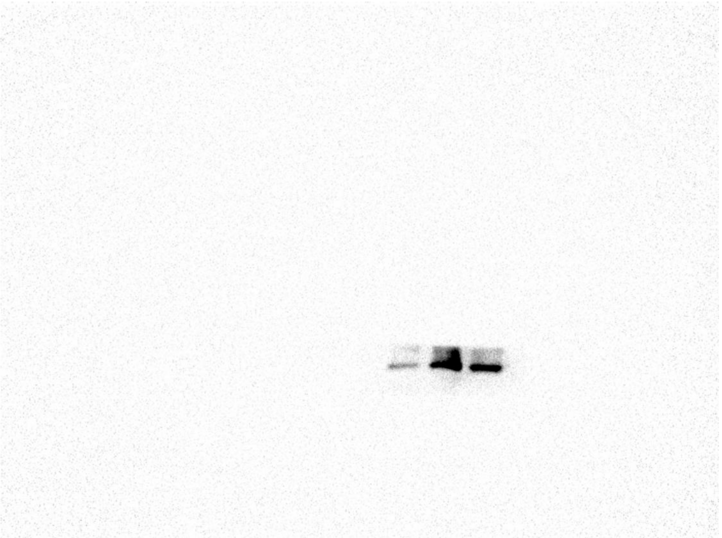

supplemental 3H-GAPDH-DU145:

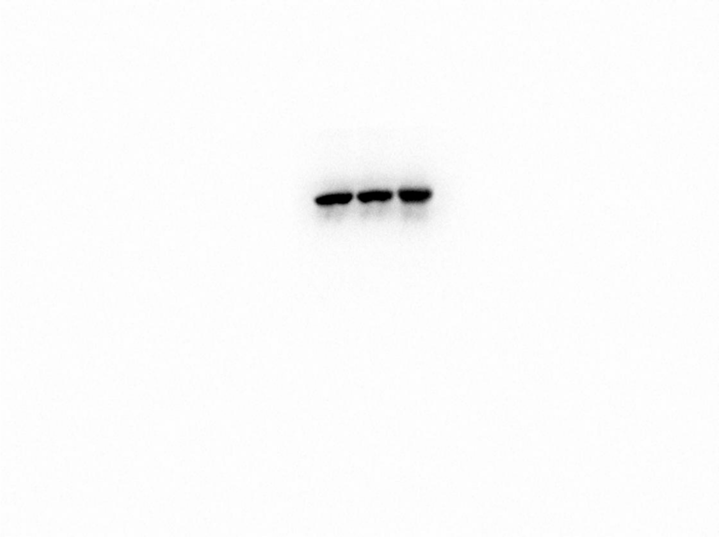

supplemental 3H-cyclinD1-PC-3:

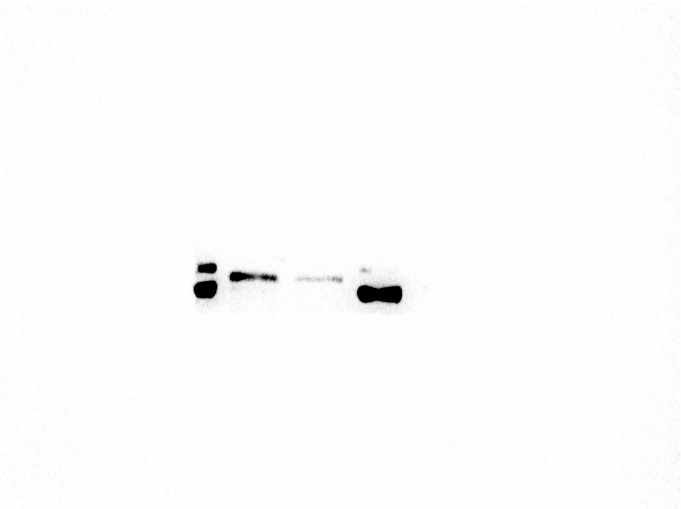

supplemental 3H-GAPDH-PC-3:

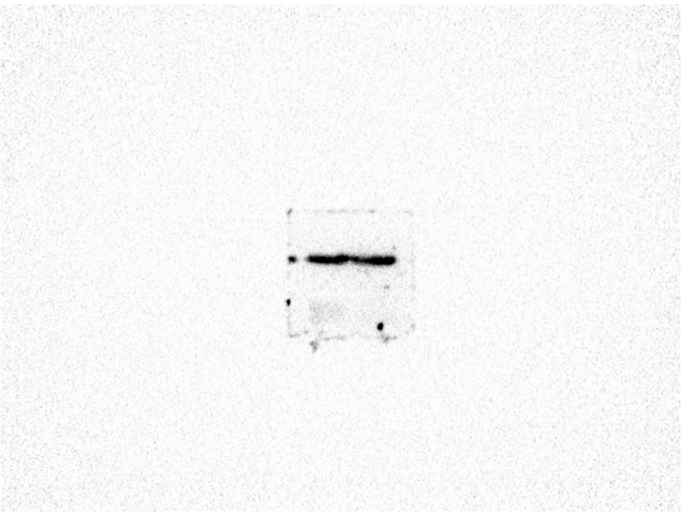

suplemental 4A-SLC7A11-DU145:

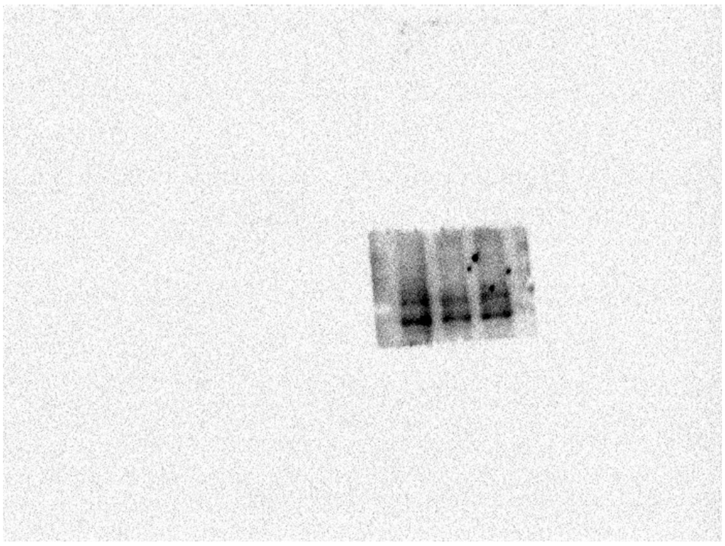

suplemental 4A-FSP1-DU145:

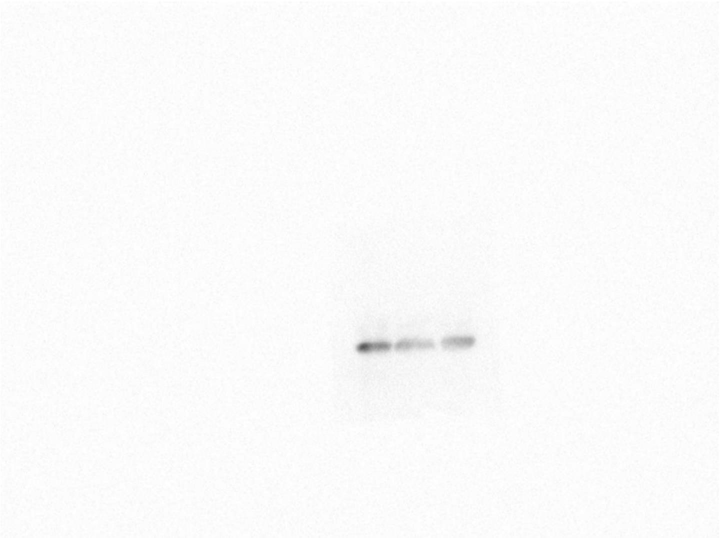

suplemental 4A-GCH1-DU145:

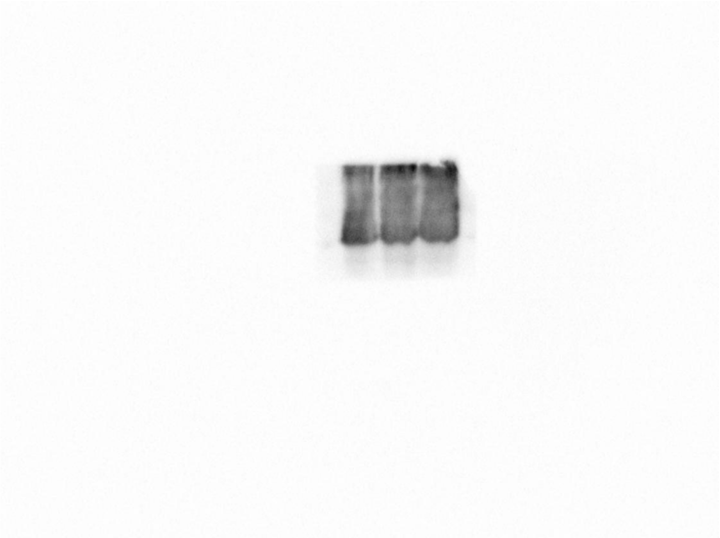

suplemental 4A-GPX4-DU145:

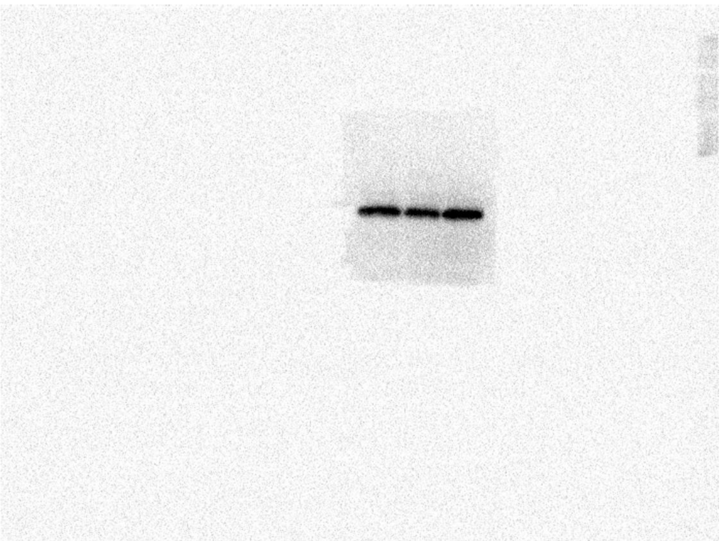

suplemental 4A-GAPDH-DU145:

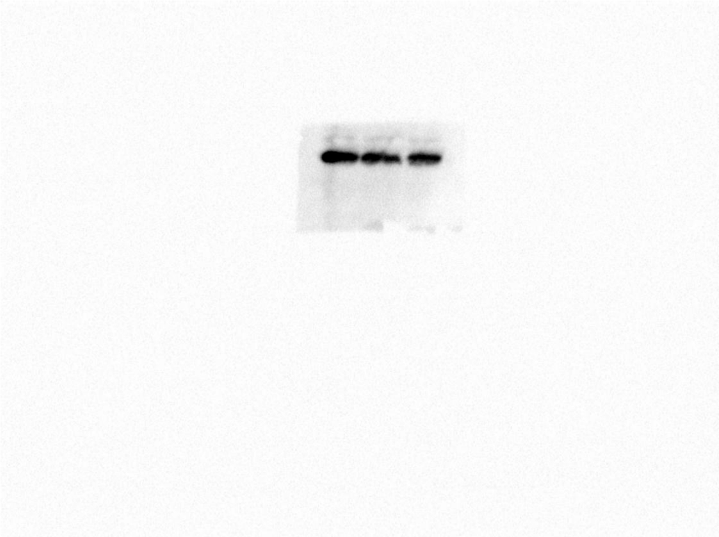
supplemental 4A-SLC7A11-PC-3:
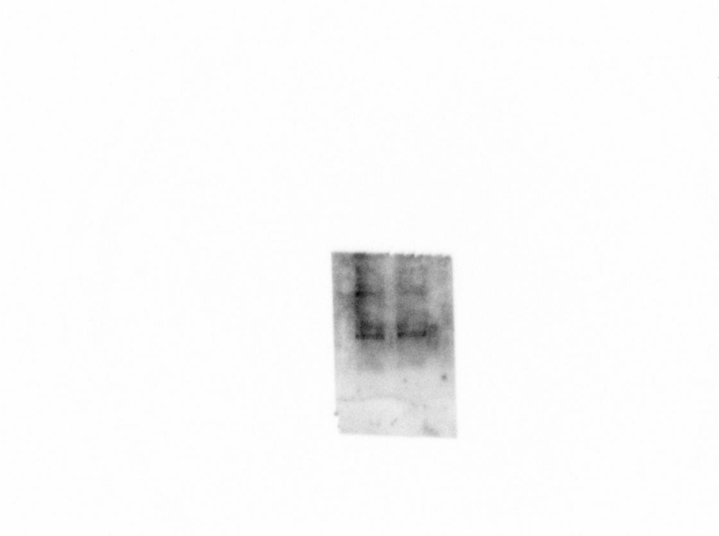
supplemental 4A-FSP1-PC-3:
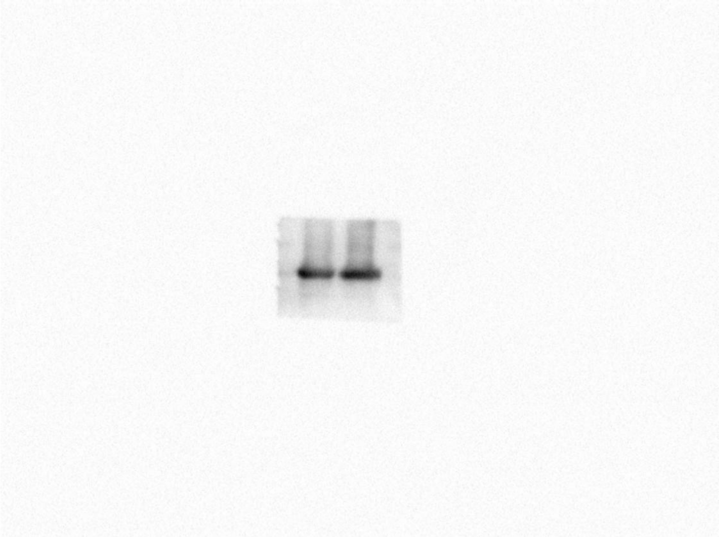
supplemental 4A-GCH1-PC-3:

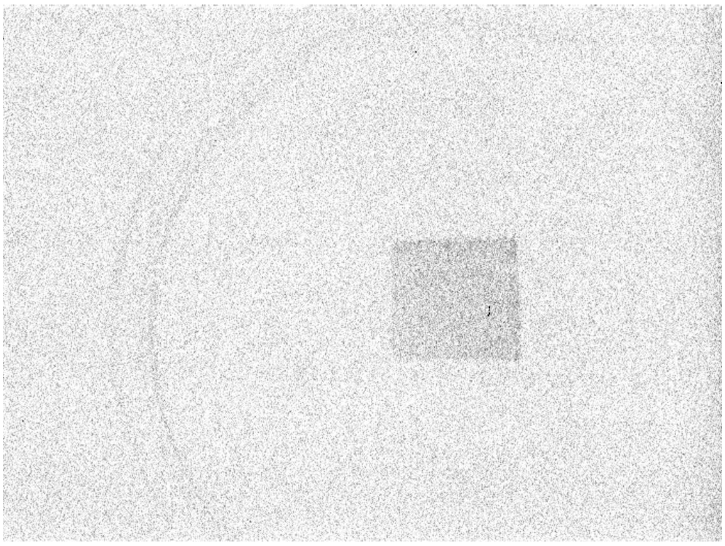

supplemental 4A-GPX4-PC-3:

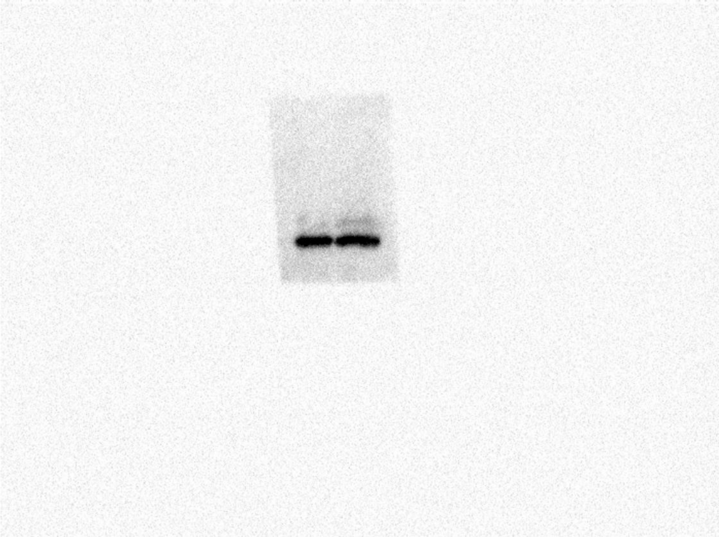

supplemental 4A-GAPDH-PC-3：

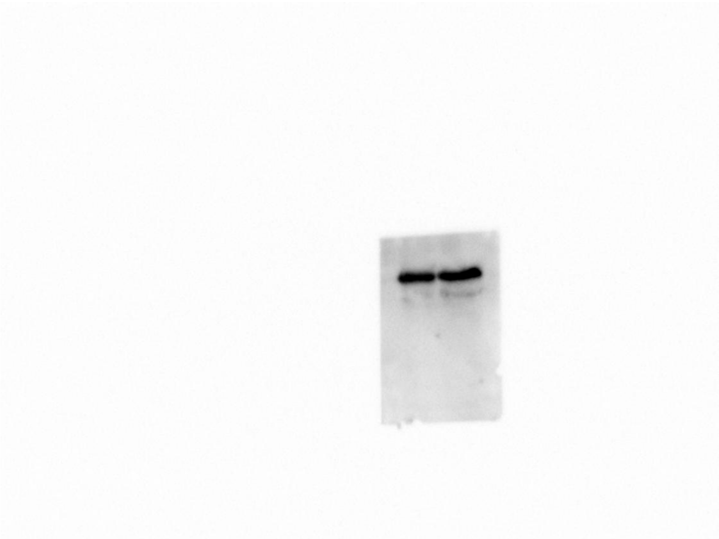

supplemental 5A-YAP-DU145（nuc）:

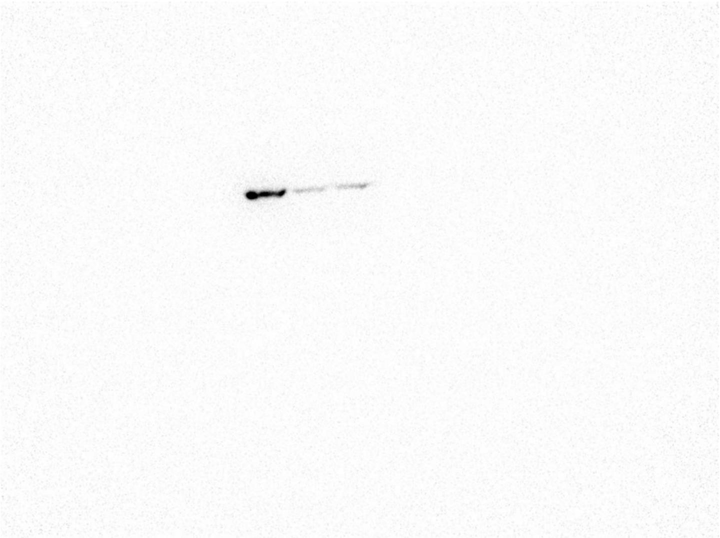

supplemental 5A-YAP-DU145（cyto）:

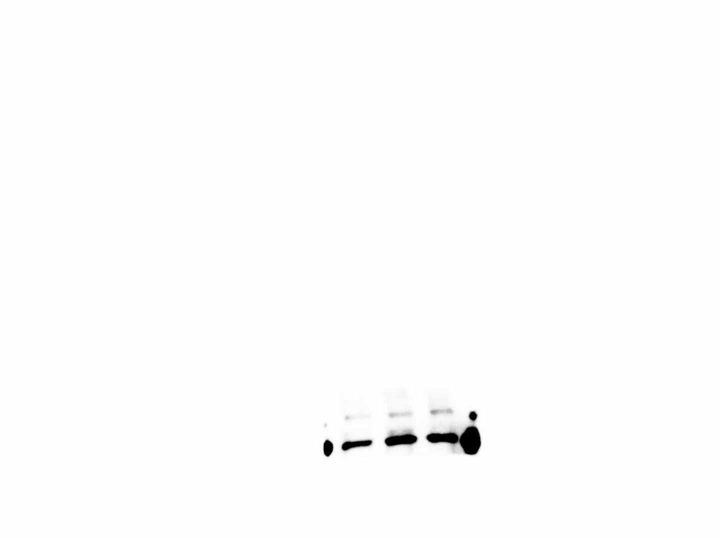

supplemental 5A-GAPDH-DU145（nuc):

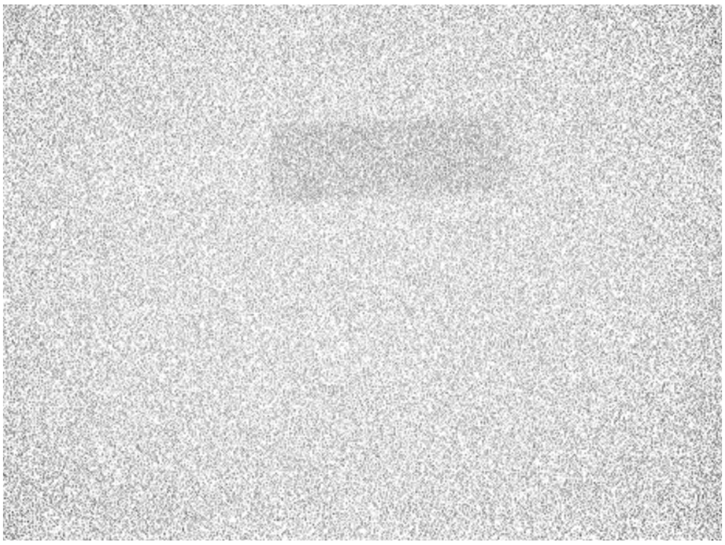

supplemental 5A-GAPDH-DU145（cyto):

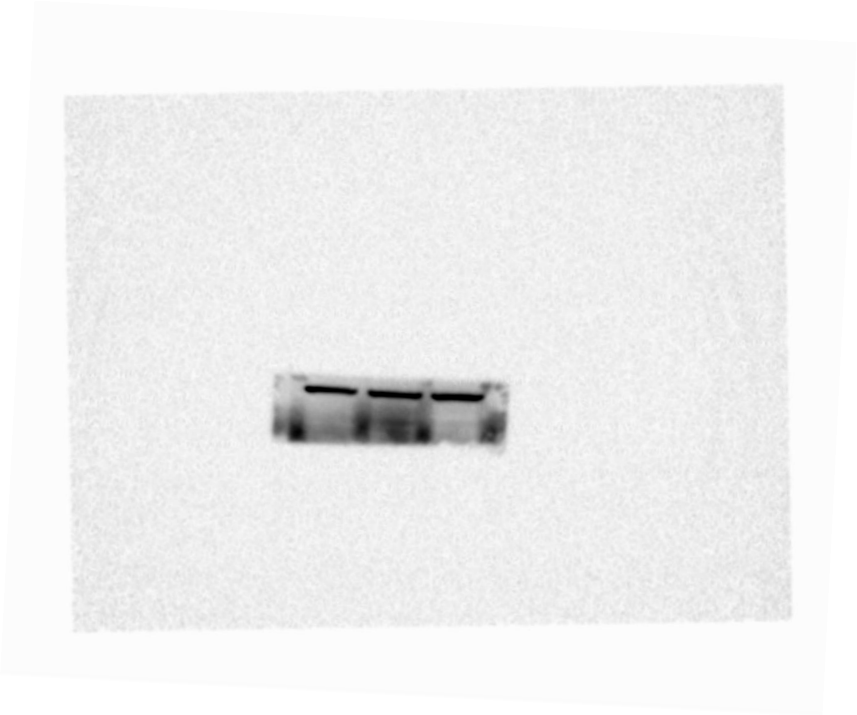

supplemental 5A-Lamin B1-DU145（cyto）:

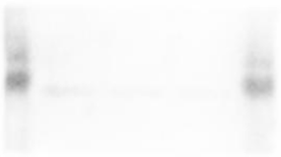

supplemental 5A-Lamin B1-DU145（nuc）:

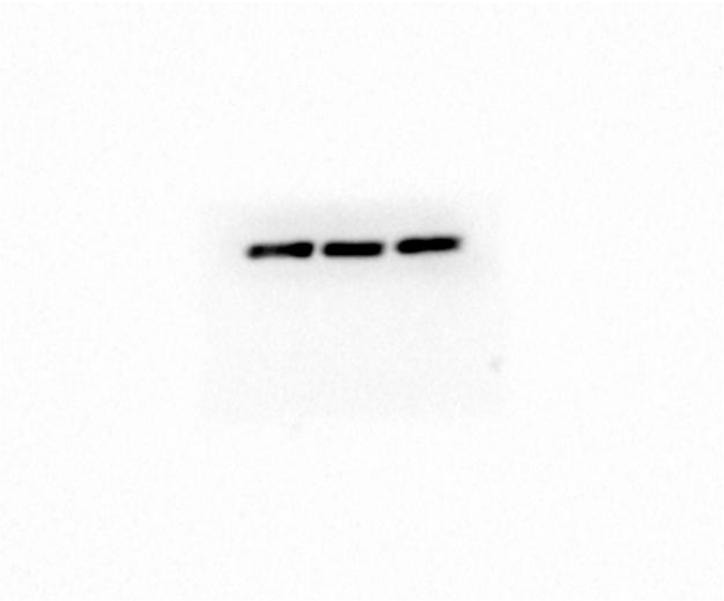

supplemental 5A-YAP-PC-3（nuc）:

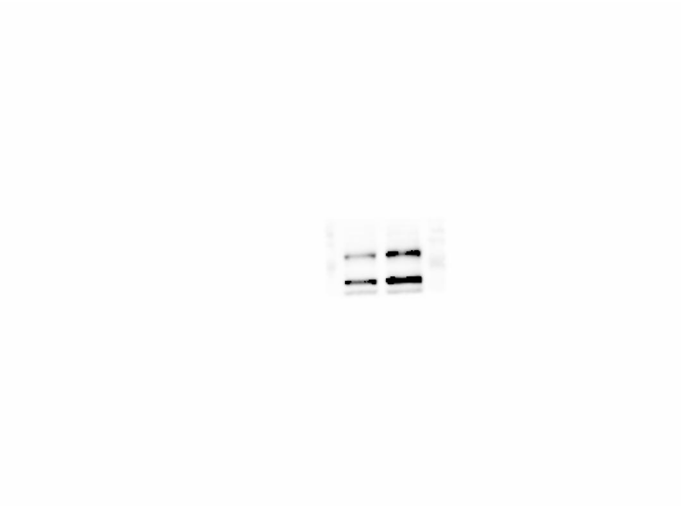

supplemental 5A-YAP-PC-3（cyto）:

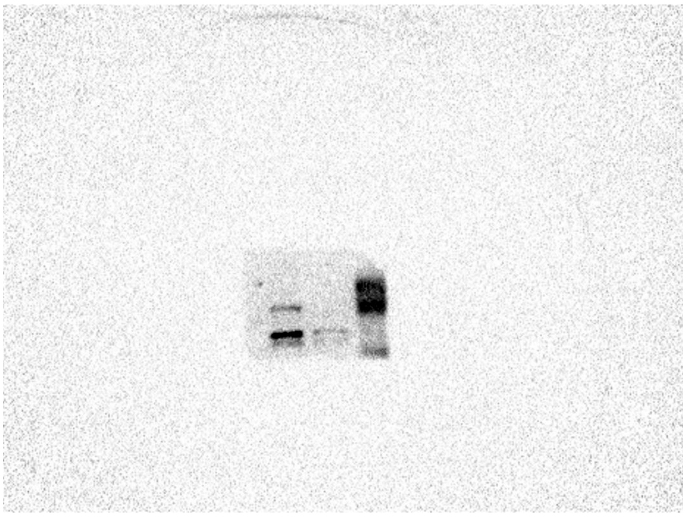

supplemental 5A-GAPDH-PC-3（cyto）:

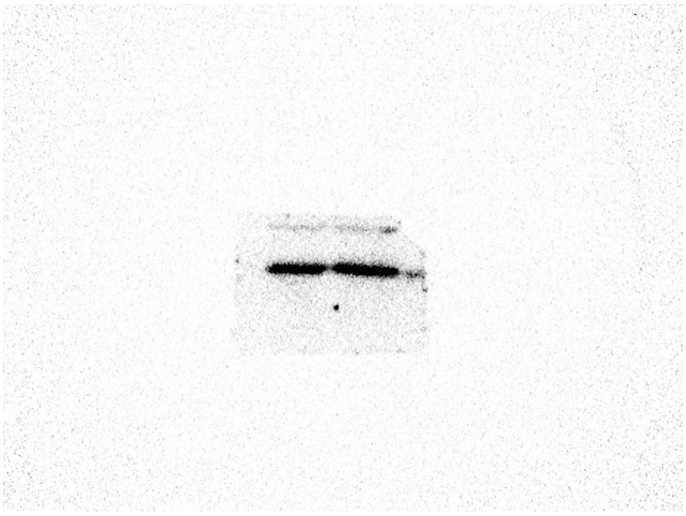

supplemental 5A-GAPDH-PC-3（nuc）:

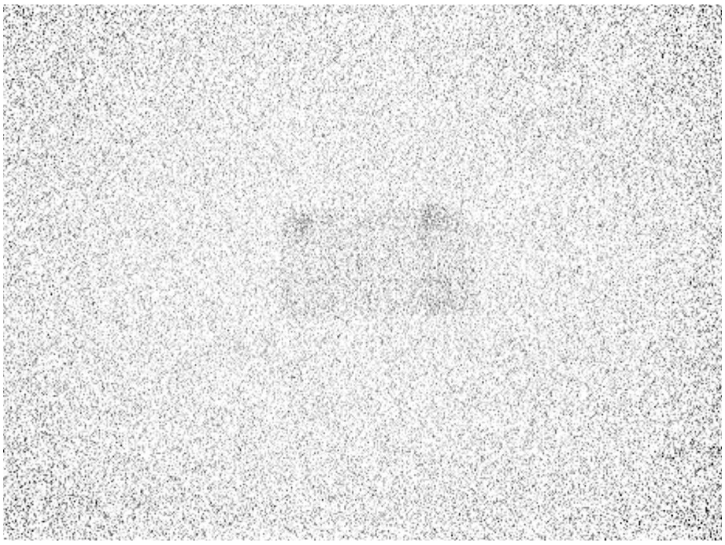

supplemental 5A-Lamin B1-PC-3（nuc）:

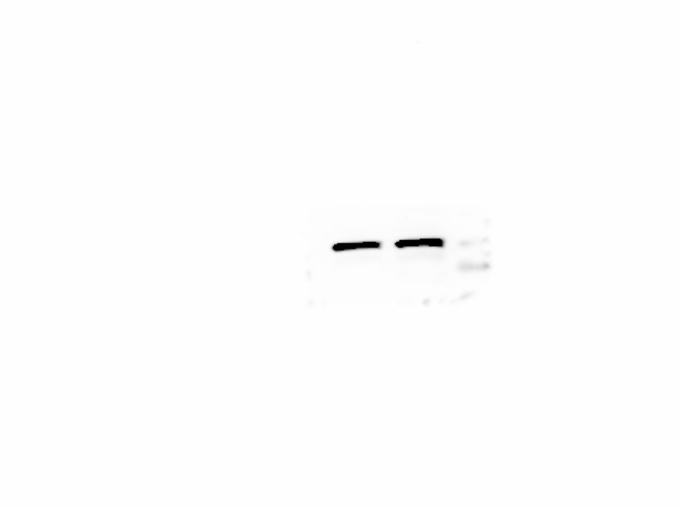

supplemental 5A-Lamin B1-PC-3（cyto）:
